# Single-cell transcriptome dissection of the toxic impact of Di (2-ethylhexyl) phthalate on primordial follicle assembly

**DOI:** 10.7150/thno.55006

**Published:** 2021-03-05

**Authors:** Jun-Jie Wang, Yu Tian, Ming-Hao Li, Yan-Qin Feng, Li Kong, Fa-Li Zhang, Wei Shen

**Affiliations:** College of Life Sciences, Key Laboratory of Animal Reproduction and Germplasm Enhancement in Universities of Shandong, Qingdao Agricultural University, Qingdao 266109, China

**Keywords:** DEHP, primordial follicle assembly, female germ cell, granulosa cell, single-cell transcriptome

## Abstract

**Rationale:** Accumulated evidence indicates that environmental plasticizers are a threat to human and animal fertility. Di (2-ethylhexyl) phthalate (DEHP), a plasticizer to which humans are exposed daily, can trigger reproductive toxicity by acting as an endocrine-disrupting chemical. In mammals, the female primordial follicle pool forms the lifetime available ovarian reserve, which does not undergo regeneration once it is established during the fetal and neonatal period. It is therefore critical to examine the toxicity of DEHP regarding the establishment of the ovarian reserve as it has not been well investigated.

**Methods:** The ovarian cells of postnatal pups, following maternal DEHP exposure, were prepared for single cell-RNA sequencing, and the effects of DEHP on primordial follicle formation were revealed using gene differential expression analysis and single-cell developmental trajectory. In addition, further biochemical experiments, including immunohistochemical staining, apoptosis detection, and Western blotting, were performed to verify the dataset results.

**Results:** Using single-cell RNA sequencing, we revealed the gene expression dynamics of female germ cells and granulosa cells following exposure to DEHP in mice. Regarding germ cells: DEHP impeded the progression of follicle assembly and interfered with their developmental status, while key genes such as *Lhx8*, *Figla,* and others, strongly evidenced the reduction. As for granulosa cells: DEHP likely inhibited their proliferative activity, and activated the regulation of cell death. Furthermore, the interaction between ovarian cells mediated by transforming growth factor-beta signaling, was disrupted by DEHP exposure, since the expression of GDF9, BMPR1A, and SMAD3 was affected. In addition, DNA damage and apoptosis were elevated in germ cells and/or somatic cells.

**Conclusion:** These findings offer substantial novel insights into the reproductive toxicity of DEHP exposure during murine germ cell cyst breakdown and primordial follicle formation. These results may enhance the understanding of DEHP exposure on reproductive health.

## Introduction

Environmental pollution is evident worldwide and is obviously threatening human health, including increasing the risk of cancer. Among various pollutants, ubiquitous plastics are annually utilized in staggeringly large volumes; this has raised a public health concern. Phthalates are widely used as a plasticizer for polyvinyl chloride resin (PVC) in the production of plastics 1. As phthalates are not covalently bond to polymers 2, 3, they can easily migrate and penetrate human and animal bodies through various routes, such as oral ingestion, inhalation, and dermal exposure 4, 5. Worryingly, human exposure to phthalate occurs on a daily basis 1, 6. Moreover, robust evidence has heightened a link between plasticizer exposure and some reproductive disorders 7-10. Thus, an investigation into the reproductive toxicity of phthalates is deemed necessary to heighten awareness of the need for protection against detrimental phthalates, and to propose potential preventive and therapeutic strategies for toxicity mitigation.

In females, primordial follicle (PF) formation (or assembly) occurs perinatally in mice and around mid-gestation in humans; this results in a finite follicle reserve termed the ovarian reserve or PF pool 11-13. After primordial germ cells arrive at the murine gonad, they undergo mitosis to produce germ cell cysts (or nests), from which oogonia mature after sexual differentiation. Subsequently, oogonia enter meiosis around 13.5 days post coitum (dpc) to become oocytes. Shortly afterwards, germ cell cysts breakdown following the invasive activity of proliferative pre-granulosa cells from 17.5 dpc to a few days postnatally. A substantial loss of germ cells, and the surviving oocytes become enclosed within a layer of flattened granulosa cells, which signifies PF formation 14, 15. It is known that the PF pool is non-renewable once established and that it progressively diminishes following the first-wave of follicle recruitment during puberty, and is almost exhausted at menopause in humans 16-18. Thus, the size of the PF pool is the primary determinant of reproductive lifespan in females 19. Notably, the available oocytes within PFs naturally undergo irreversible exhaustion or atresia during development; however, they are prone to damage from adverse external environmental chemicals (such as plasticizers) as well as cellular aging 14, 20. Meanwhile, the ovarian dysgenesis syndrome hypothesis proposes that an early disruption to ovarian development can potentially lead to ovarian disease and disorders in adulthood; this is particularly pertinent at times that are sensitive to disruption by exogenous chemicals, such as PF assembly 21-23. Consequently, attention needs to be focused on the protection of female fertility from toxic materials.

Di (2-ethylhexyl) phthalate (DEHP), the most commonly used plasticizer, is known to be an endocrine-disrupting chemical (EDC) that can trigger reproductive and developmental toxicity in females 1, 21. Importantly, DEHP exposure impairs establishment of the ovarian reserve by delaying or reducing PF assembly 24, 25. Similar observations in other studies suggest the deregulation of estrogen receptor (ER) and progesterone receptor expression, and attenuation of NOTCH signaling 26. Furthermore, the number of apoptotic germ cells increases during *in vitro* culture of newborn ovaries exposed to DEHP for three days 25. Specifically, autophagy can be induced by DEHP exposure via activating AMPK-SKP2-CARM1 signaling, thus further reducing the PF pool and damaging female fertility 27; these results indicate that DEHP affects oocyte survival during PF formation. From another aspect, *in vivo* and *in vitro* studies both suggest that DEHP damages steroidogenesis and exerts toxicity during different stages of folliculogenesis 9, 28. For example, plasticizer exposure could accelerate the development of PFs and therefore result in premature ovarian failure (POF) 29, and antral follicle depletion is increased as a result of estrogen deficiency, estrous cycle disorder, and impaired aromatase activity 4, 7, 30, 31. Meanwhile, the transgenerational inheritance of DEHP has attracted considerable attention 32-34. Through maternal exposure, DEHP has been shown to exert adverse reproductive effects across generations: it reduces oocyte quality and follicle reserve in F1 female mice, and alters the same ovarian phenotype in F2 and F3 offspring; moreover, it accelerates follicular recruitment 24, 35.

The development of single-cell transcriptomics has revolutionized the dissection of cellular heterogeneity and cell fate transition 36-38. From such studies, many novel insights have been proposed regarding reproductive system development, derived from single cell datasets in both males 39-42 and females 43-46. Recently, the credo of a limited ovarian reserve has been reinforced by single-cell analysis of human ovarian cortical tissue, where no oogonial stem cells were detected 47. In addition, the transcriptomic landscapes of germ cells during early murine oogenesis have been delineated, which concentrates on two vital germline events, meiotic initiation 43, 48 and PF assembly 49. Furthermore, the respective waves of PF formation in the ovarian cortex and medulla disclose two distinct pathways for pre-granulosa cell differentiation 50. To date, the effect of DEHP on PF assembly has not been thoroughly examined and deciphered.

The current study set out to characterize the molecular mechanism of the toxic effects of DEHP exposure on PF assembly, at a daily dose of 40 µg/kg body weight DEHP through oral administration to pregnant mice at 16.5 dpc. Subsequently, the DEHP-treated ovaries at PD0 (PD0-DEHP) and PD3 (PD3-DEHP) were subjected to single-cell RNA sequencing (scRNA-seq) using a 10× Genomics platform. At a single-cell level, the toxicity of DEHP exposure to PF assembly was further investigated, and compared to previously reported normal murine developmental ovarian datasets at PD0 and PD3 49.

## Results

### scRNA-seq identified six main cell types in post-natal ovaries

**Figure 1:**
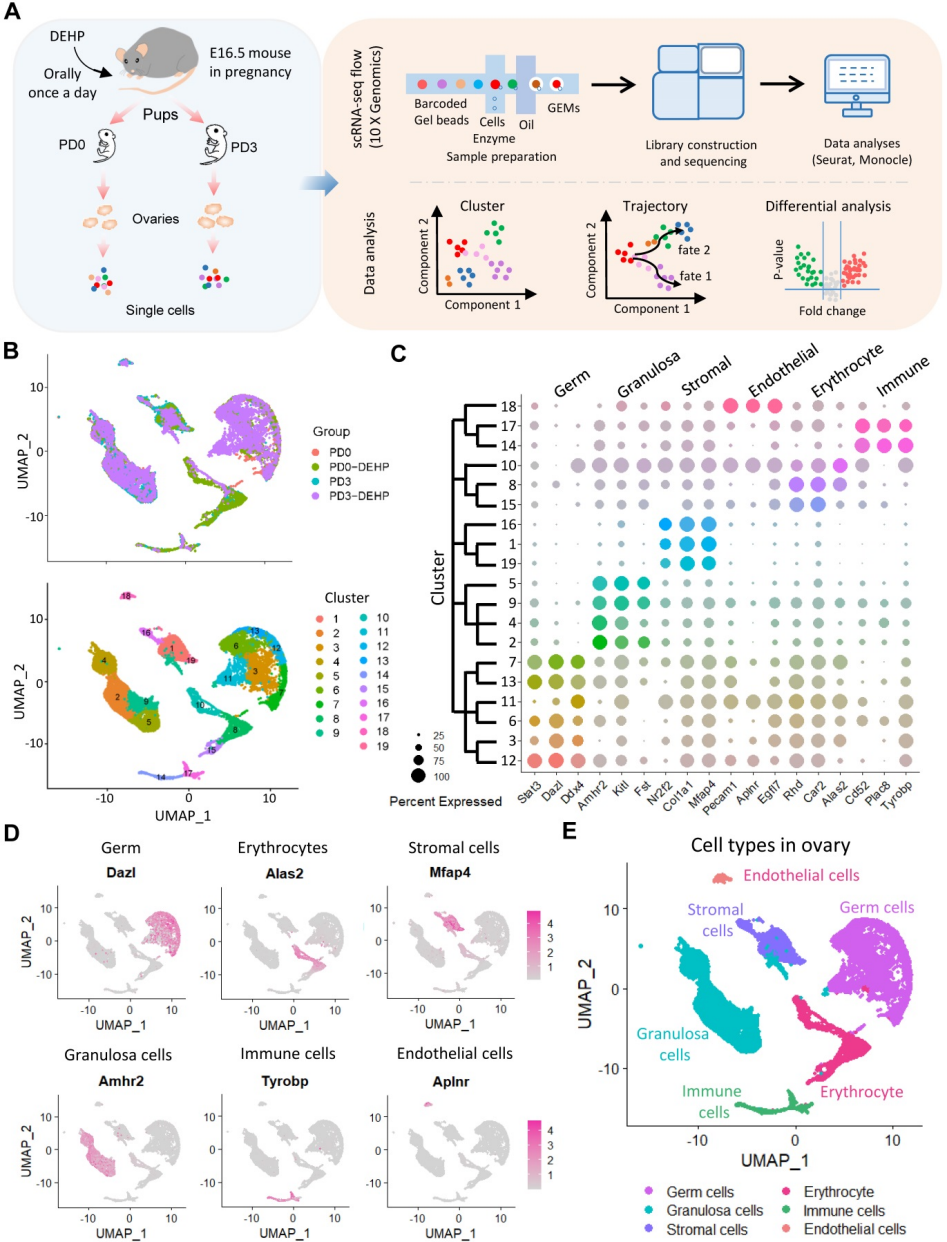
**Molecular characterization of post-natal ovaries. (A)** A schematic pipeline of sample collection, 10× Genomics platform sequencing, and data analysis. **(B)** UMAP plots of ovarian cells based on sample group (upper) and Seurat cluster (below). **(C)** Dot plot of the three marker genes identified from ovarian cell types in each cluster. Dot size represents the percentage of gene expression in each cell cluster; color intensity refers to the intensity of expression, with strong color indicating high gene expression. The dendrogram of cell clusters is presented in the left panel. **(D)** Feature plots of specific marker genes from six main ovarian cell types. **(E)** The six main ovarian cell types are identified on a UMAP plot.

To characterize the transcriptome dynamics of ovarian cells affected by DEHP during PF formation, pregnant mice were given DEHP daily from 16.5 dpc, and the ovaries were collected at postnatal day 0 (PD0-DEHP) and 3 (PD3-DEHP), from which single-cell suspensions were prepared for scRNA-seq (Figure 1A, left panel). Following the combination of barcoded gel beads, enzyme mixtures, and oil, the nanoliter-scale droplets (termed “gel bead-in emulsions”, GEMs) were generated for library construction and sequencing (upper part of right panel). Then, single-cell datasets were conducted for cell cluster, pseudotime trajectory, and differential analysis of gene expression using Seurat and Monocle (Figure 1A, bottom part of right panel). In addition, after data pre-progressing and potential doublets filtration (Figure S1A), 4,558 high-quality ovarian cells were obtained at PD0-DEHP and 5,252 at PD3-DEHP, and the number of high-quality cells from collected datasets of normal ovarian tissues at PD0 was 4,270, with 5,375 at PD3 (Figure S1B). The harvested high-quality cells were used for subsequent analysis.

Based on uniform manifold approximation and projection (UMAP) technology, 19 cell clusters were generated (Figure 1B), and several cell clusters were at least partially displayed in a dependent manner for the developmental stage or DEHP treatment. To determine the ovarian cell composition at an investigated period, marker genes were identified in cell clusters (Table S1); the top five marker genes in each cluster are shown in Figure S1C. According to their transcription characteristics, cell clusters were divided into six cell types, and the representative genes in each cell type are shown in Figure 1C. In addition, the cell-type-specific genes are presented in Figure 1D and Figure S1D; six main cell types were identified (Figure 1E). Moreover, germ cells and granulosa cells were examined as the major participants of PF assembly using immunohistochemistry; STAT3 for germ cells (Figure S2A) and AMHR2 for granulosa cells (Figure S2B), respectively.

### DEHP impacted the progress of PF assembly and germ cell development

**Figure 2:**
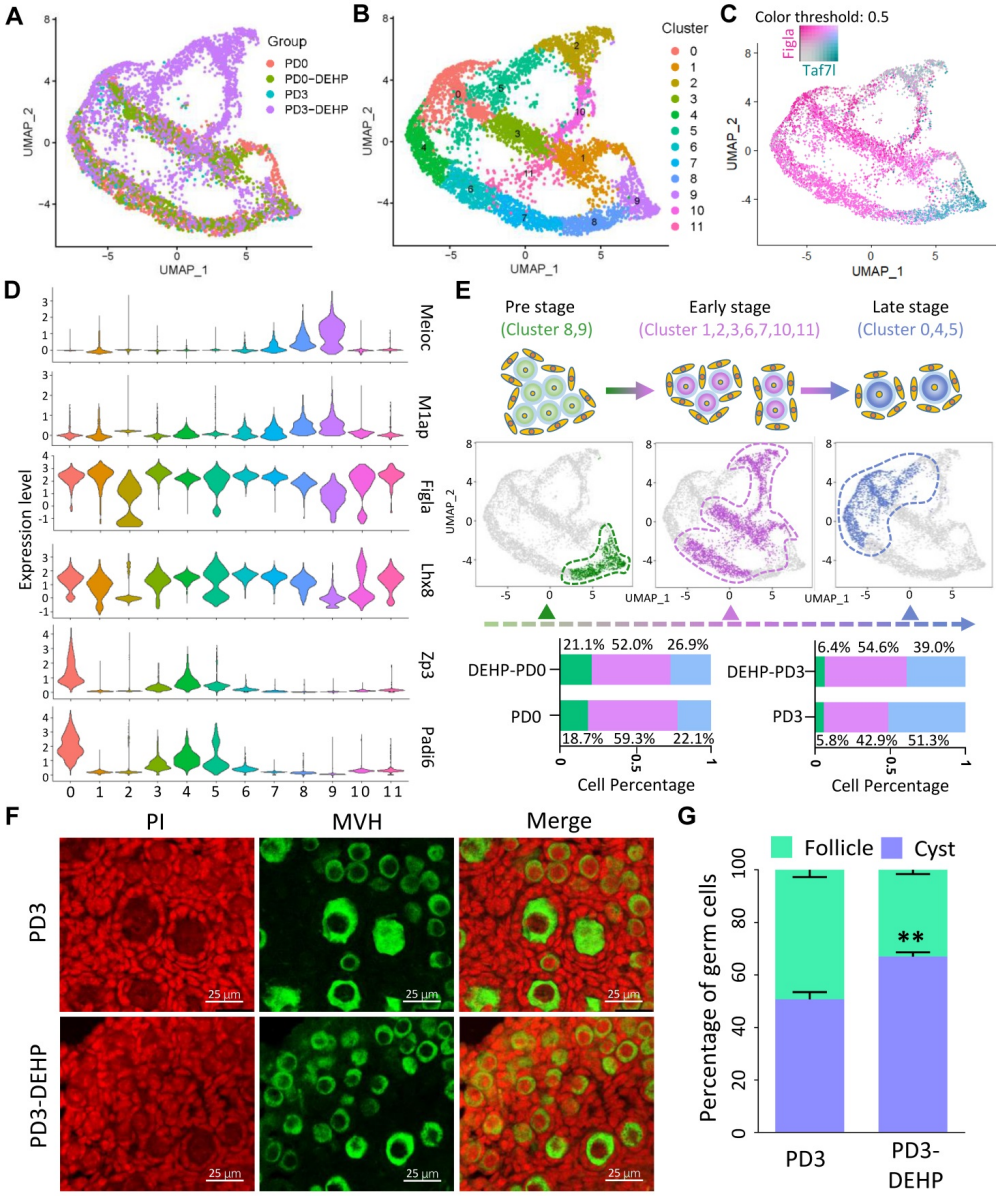
**DEHP impacted the progress of germline cyst breakdown and follicular assembly. (A)** Clustering of germ cell population with UMAP, colored based on sample groups. **(B)** Clustering of germ cell population with UMAP, colored based on 12 cell clusters. **(C)** Expression of *Taf7l* and* Figla* separating the germ cell clusters into different developmental stages based on UMAP. The color intensity of cyan or maroon represents gene expression level; grey refers to the double-negative cells and the color threshold of gene expression is 0.5. **(D)** Expression of representative genes in cell clusters for germ cell-specific developmental stages, the pre-stage of follicle formation with highly expressed meiosis related genes: *Meioc, M1ap*; the early-stage marked by high expression of key genes for follicle formation: *Figla, Lhx8*; the late-stage contained genes required for oocyte growth: *Zp3, Padi6*. **(E)** The three separated developmental stages in germ cell clusters. Cell cluster classification, feature plots and cell percentages of sample groups for the three stages are shown in the upper, middle, and bottom panels. **(F)** Representative images of ovarian sections with normal and DEHP-treated groups at PD3. Germ cells were marked by MVH (green) and cell nuclei were counterstained with propidium iodide (PI, red). Scale bar = 25 μm. (G) The percentages of germ cells within cysts and follicles at PD3 with and without DEHP exposure. Data are presented as mean ± SD (N = 3 for independent repeats, the total germ cell counting at PD3 in number was 1,710, 1,355, and 1,401, and 2,004, 1,828, and 2,341 at PD3-DEHP). **P < 0.01.

Following the cell type classification above, the germ cell population was extracted for in-depth analysis; according to the results, cell heterogeneity was not dependent on cell cycle genes (Figure S3A) and cells in the four groups were classified as twelve cell clusters (Figure 2A-B). In addition, marker genes in each germ cell cluster were identified (Table S2), and the top five genes are displayed in a heatmap in Figure S3B. Among them, several key genes involved in biological events were highlighted, including meiosis (*Meioc, M1ap*), PF assembly (*Figla, Lhx8*), and oocyte growth (*Zp3, Padi6*; Figure S3C). Specifically, the representative genes, *Taf7l* and *Figla*, were capable of separating cell clusters into two developmental stages (Figure 2C). Meanwhile, based on the expression pattern of genes associated with meiosis, PF assembly, and oocyte growth in clusters (Figure 2D and Figure S3C), the germ cells were divided into three developmental stages: pre-, early-, and late-stage of PF assembly (Figure 2E). The pre-stage consisted of cells in clusters 8 and 9; the early-stage contained cells in clusters 1, 2, 3, 6, 7, 10, and 11; and the late-stage comprised cells in clusters 0, 4, and 5 (Figure 2E upper). Moreover, the percentage of cells at the three stages indicated a decline in cell percentage at the early-stage for PD0 and the late-stage for PD3 after DEHP treatment (Figure 2E bottom); furthermore, the results suggested that DEHP impacted the progress of PF formation. In particular, the immunostaining of ovarian sections at PD3 (Figure 2F) with or without DEHP suggested that DEHP exposure indeed decreased the percentage of germ cells within follicles (Figure 2G).

**Figure 3:**
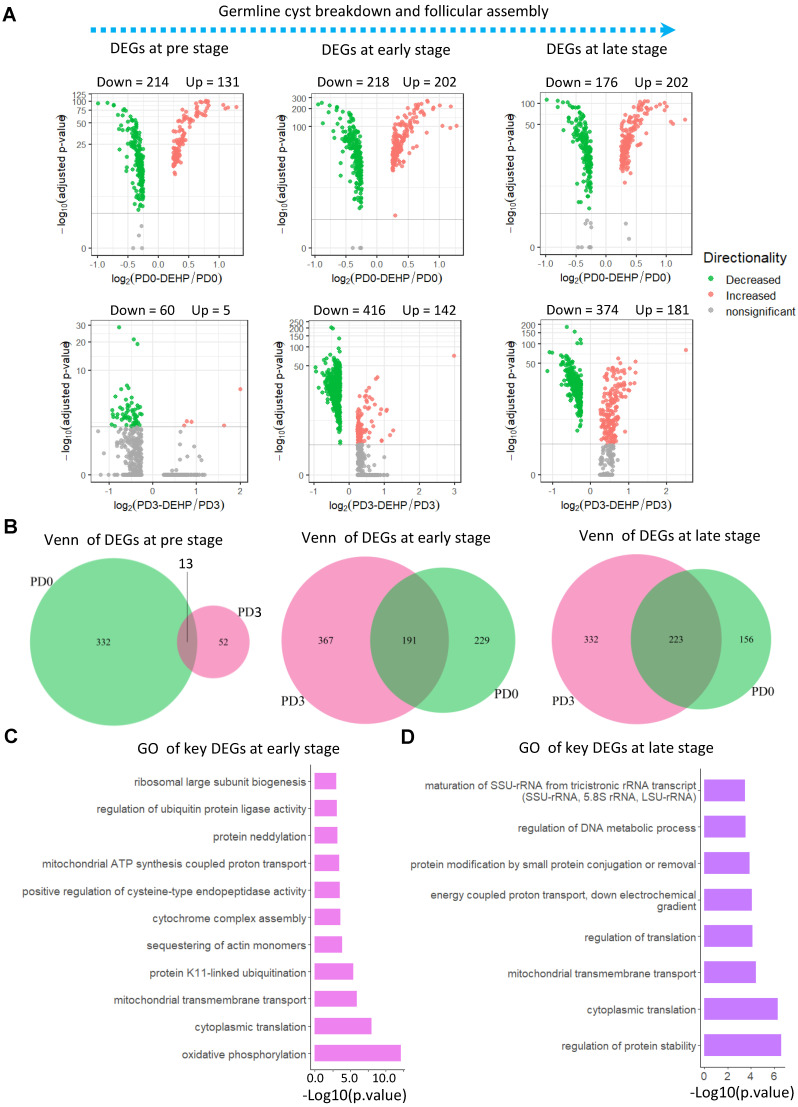
**Differential expression of stage-specific germ cells with or without DEHP treatment. (A)** Volcano plots of germ cells with three developmental stages. The left panel illustrates differentially expressed genes (DEGs) of germ cells at the pre-stage of PF formation with or without DEHP treatment for PD0 and PD3, respectively. The middle and right panels refer to pre- and late-stage. **(B)** Venn plots of germ cells with three developmental stages. The light green and violet red circles represent the number of DEGs at PD0 and PD3. **(C-D)** GO terms of key DEGs at early- (C) and late-stages (D) of PF formation.

Furthermore, gene differential expression was carried out at the three identified stages (Figure 3A). At the pre-stage of PF formation (Table S3), there were 214 down- and 131 up-regulated genes for PD0-DEHP *vs.* PD0, and 65 differentially expressed genes (DEGs) between the PD3-DEHP and PD3 groups (Figure 3A left). For the early-stage (Table S4) with DEHP treatment, there were 202 up- and 218 down-regulated DEGs at PD0, and 142 up- and 416 down-regulated DEGs at PD3, respectively (Figure 3A middle). At the late-stage (Table S5), DEHP significantly increased 202 and 181 DEGs, and decreased 176 and 374 genes at PD0 and PD3, respectively (Figure 3A right). Furthermore, the representative genes involved in PF assembly and oocyte growth dramatically declined (Figure S3D). These results implied that DEHP tended to influence the transcription of germ cells during PF assembly. In addition, 13, 191, and 223 key DEGs (this refers to the common genes in Venn diagrams that were both affected by DEHP at PD0 and PD3) were produced at the pre-, early-, and late-stages of PF formation, respectively (Figure 3B). Enrichment analysis demonstrated that key genes at the pre-stage were related to “Ribosome”, “DNA repair”, and “Regulation of cellular response to stress” (Figure S3E); 191 key DEGs at the early-stage were mostly enriched in the biological processes of “oxidative phosphorylation”, and “mitochondrial ATP synthesis coupled proton transport” (Figure 3C). Meanwhile, at the late-stage, DEHP largely affected “regulation of protein stability” (Figure 3D), as well as “energy coupled proton transport”. Moreover, both were enriched in “cytoplasmic translation” and “mitochondrial transmembrane transport”.

In a separate analysis, the DEG lists at PD0 and PD3, that were associated with the DEHP effect, were examined. Results showed that at the pre-stage, the biological process of DEG lists at PD0 and PD3 varied massively (Figure S4A), and the difference at the early- and late-stages of PF formation were not distinct (Figure S4B-C). Furthermore, DEHP always impacted the processes of “cellular responses to stress”, “chromatin organization”, “oxidative phosphorylation”, “cytoplasmic translation”, “cellular response to DNA damage stimulus”, and “transcriptional regulation by TP53” throughout both PD0 and PD3 stages. At the same time, the transforming growth factor-beta (TGF-beta) signal, cell cycle, and mRNA processing were all likely associated with DEHP effect on germ cell development.

### DEHP disordered germ cell fate revealed by single cell trajectory

**Figure 4:**
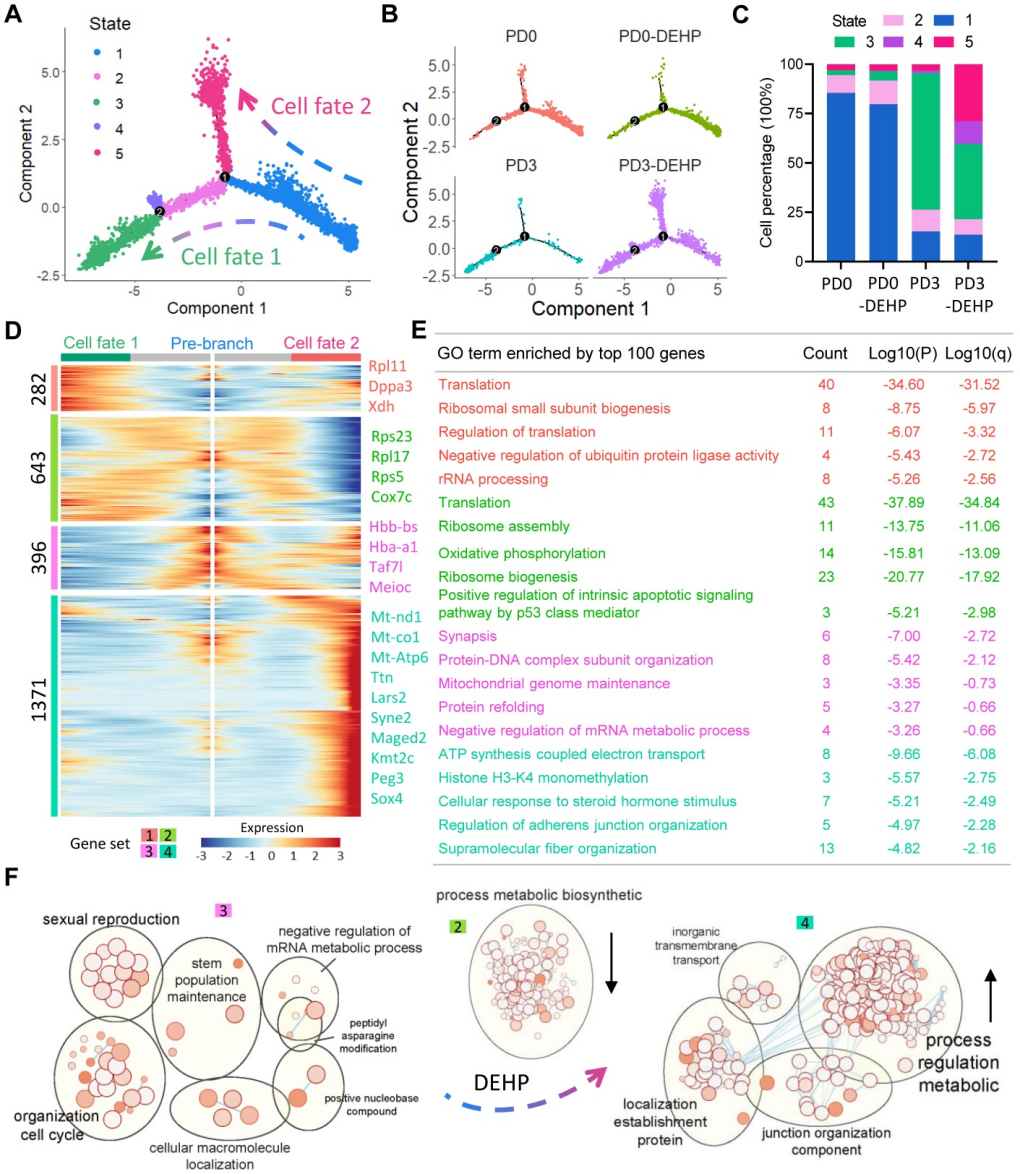
**DEHP influenced the developmental status of germ cells during PF formation. (A)** The pseudotime trajectory of germ cell populations colored by five States. Three branches were identified with two developmental fates. **(B)** The pseudotime trajectory of germ cell population colored by four sample groups. **(C)** Cell percentages of five States among four sample groups. **(D)** Pseudotime ordered heatmap of four differentially expressed gene sets between two obvious fates at branch point one. Several representative genes of each gene set are listed in the right panel. **(E)** The enrichment of GO terms of the top 100 genes in each gene set. **(F)** Enrichmentmap illustrated that DEHP exposure disturbed multiple processes in germ cells.

To further dissect the fate transition of germ cells during PF formation, the pseudotime trajectory of germ cells was established to reveal five developmental States and two branching cell fates (Figure 4A). According to the distribution of germ cells in States, the differentiated status of DEHP-treated germ cells at PD3 underwent a remarkable change, with an apparent increase of cells at State 5 being observed (Figure 4B-C). Gene expression along with cell trajectory showed at State 1 that the *Taf7l* gene was actively expressed at the pre-stage of PF formation, while the *Figla* gene was expressed at all investigated stages, while at State 3 the *Zp3* and *Padi6* genes were specifically and actively expressed in follicular oocytes (late-stage; Figure S5A). This evidence implied that PF assembly was impeded by DEHP.

To investigate the inherent mechanism, gene expression patterns at two branches was performed (Figure 4D). Four gene sets regarding two cell fates were generated: set 1 consisted of 282 genes, mostly and highly expressed in cell fate 1, partly expressed at cell fate 2, and slightly expressed at pre-branch (State 1); furthermore, the GO terms of the top 100 genes in the set were mostly enriched in biological processes such as “Translation”, “Ribosomal small subunit biogenesis”, “Regulation of translation”, “Negative regulation of ubiquitin protein ligase activity” and “rRNA processing”. Set 2 contained 643 genes that were mainly expressed in pre-branch and fate 1 cells, and hugely decreased in fate 2, which acted on “Translation”, “Ribosome assembly”, “Oxidative phosphorylation”, “Ribosome biogenesis”, and “Positive regulation of intrinsic apoptotic signaling pathway by p53 class mediator”. For the 396 genes in set 3, they were mainly expressed at pre-branch, showing a slight decline at fate 2 and were dramatically down-regulated at fate 1, but largely enriched in “Synapsis”, “Protein-DNA complex subunit organization”, “Mitochondrial genome maintenance”, “Protein refolding”, and “Negative regulation of mRNA metabolic process”. The 1,371 genes in set 4 seemed to be elevated by DEHP at fate 2 and were related to the processes of “ATP synthesis coupled electron transport”, “Histone H3-K4 monomethylation”, “Cellular response to steroid hormone stimulus” and “Supramolecular fiber organization” (Figure 4E). Therefore, following DEHP exposure during PF formation, these results suggest that some biological consequences (such as “organization cell cycle” and “sexual reproduction”) persisted from pre-branch (Figure 4F, set 3), the “process metabolic biosynthetic” decreased (set 2), and “process regulation metabolic” increased (set 4). Moreover, the representative genes in each gene set are shown in Figure S5B; for set 4, with enrichment analysis of protein-protein interaction, the genes seemed to be involved in “chromatin organization”, “covalent chromatin modification”, and “histone modification” (Figure S5C).

Another noteworthy branch point existed in germ cell trajectory. In the comparative analysis, the heatmap of gene expression dynamics from State 4 to State 3 or 1 comprised two large gene sets (Figure S5D): one of known meiosis-related genes that were highly marked and expressed in State 1, such as *Taf7l*,* Sycp1*,* Sycp3,* and *Smc1b*; while the other included more key genes (*Dppa3, Zp3,* and* Padi6*) critical to PF assembly. Importantly, the gene expression level along with pseudotime indicated that State 4 was almost equidistant between States 1 and 3 (Figure S5E), therefore, the cells at State 4 appeared to be a transitional stage of female germ cells between cyst and follicle.

### DEHP impacted the development of pre-granulosa cells at the prenatal stage

**Figure 5:**
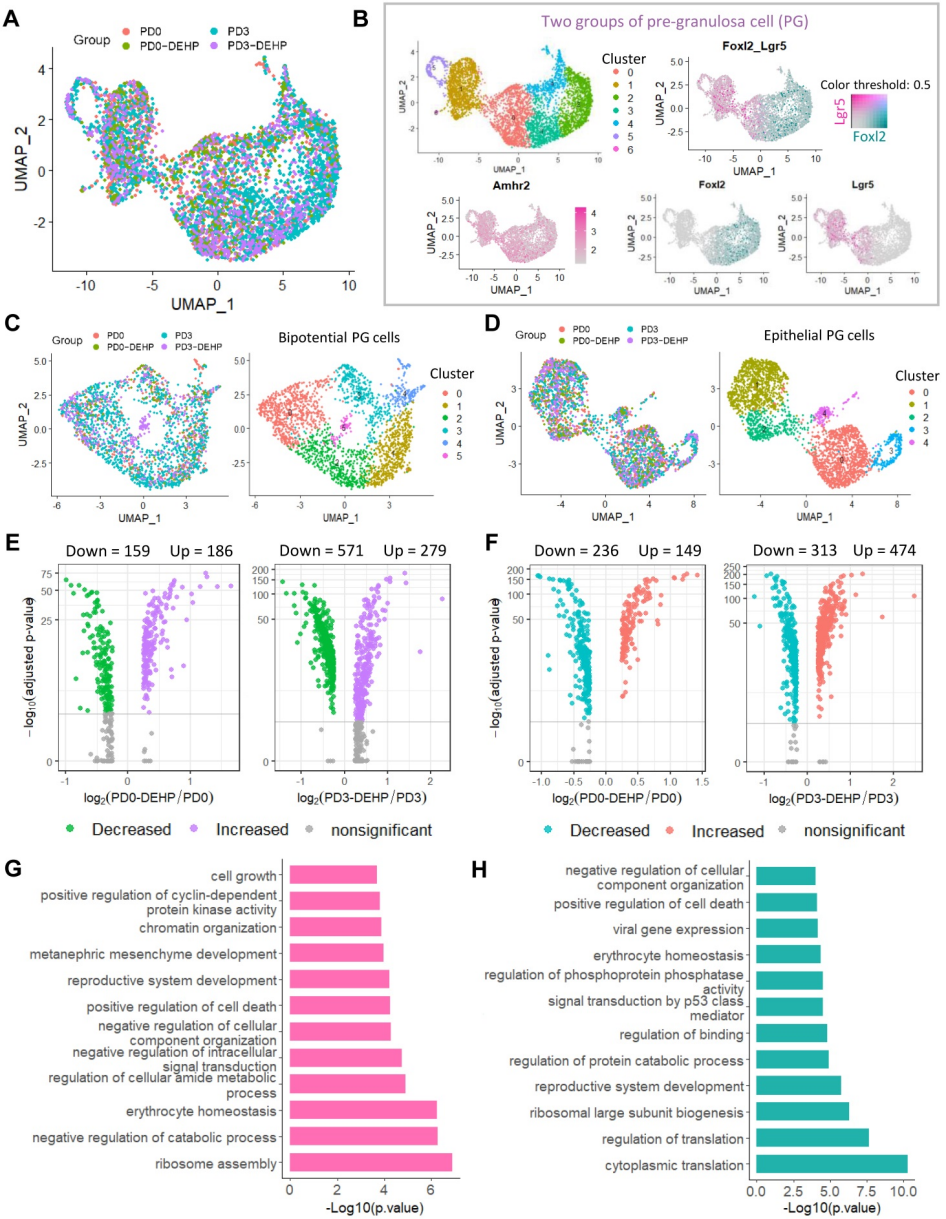
** DEHP impacted transcriptome characteristics of two groups of PGs during follicular assembly. (A)** Clustering of pre-granulosa cells based on UMAP colored by the sample groups. **(B)** Two groups of PGs. Seven clusters with high *Amhr2* expression (common marker gene of granulosa cell) are identified as PGs in the left panel; two groups of PGs are separated by the expression of *Foxl2* (cyan) and *Lgr5* (maroon). The color intensity represents gene expression level; grey refers to the double-negative cells with color threshold of gene expression at 0.5. **(C)** UMAP plot of bipotential PGs (BPGs) colored by sample groups (left) and cell clusters (right). **(D)** UMAP plot of epithelial PGs (EPGs) colored by sample groups (left) and cell clusters (right). **(E)** Volcano plots of gene differential expression at BPGs between pairs of PD0-DEHP *vs.* PD0 and PD3-DEHP *vs.* PD3. **(F)** Volcano plots of gene differential expression of EPGs between PD0-DEHP *vs.* PD0 and PD3-DEHP *vs.* PD3. **(G)** The GO term enrichment of key DEGs in BPGs. **(H)** The GO term enrichment of key DEGs in EPGs.

It is already known that pre-granulosa (PG) cells are required for PF formation. PG cells were clustered based on UMAP (Figure 5A). Given that the occurrence of two waves of ovarian follicles is supported by two distinct pathways of PG cells 44, 50, the PG cells in this study were divided into bipotential (BPG) and epithelial (EPG) types (Figure 5B). There were seven clusters of PG cells that showed a high expression of *Amhr2* in the study, which were distinguished by *Foxl2* and *Lgr5*, the marker genes of BPG and EPG cells, respectively (Figure 5B, right panel). Further, the clustering of BPG and EPG cells were separated, and six cell clusters for BPG and five for EPG were produced (Figure 5C-D); their expression patterns were consistent with previous research (Figure S6A-B) 50.

Gene differential expression analysis was applied to uncover the effects of DEHP exposure to PG cells. In BPG cells, exposure to DEHP significantly increased 186 genes and decreased 159 genes at PD0, while the number of DEGs up-regulated by DEHP at PD3 reached 279 and with 571 down-regulated (Figure 5E, Table S6). Similarly, for EPG cells, 149 and 474 genes were up-regulated by DEHP at PD0 and PD3, respectively, and 236 and 313 genes were down-regulated (Figure 5F, Table S7). In addition, 192 key DEGs in BPG and 248 key DEGs in EPG were affected by DEHP both at PD0 and PD3, respectively (Figure S6C-D). GO enrichment analysis indicated that the key DEGs of BPG cells were involved in the processes of “ribosome assembly”, “catabolic process”, “positive regulation of cell death”, and “reproductive system development” (Figure 5G). Meanwhile, genes of EPG cells were enriched in “cytoplasmic translation” and “regulation of transition”, as well as “positive regulation of cell death” and “reproductive system development” (Figure 5H). Furthermore, Metascape enrichment analysis of protein-protein interaction revealed that the key DEGs of BPG were enriched in terms of “regulation of cyclin-dependent protein kinase activity” and “positive regulation of cell cycle” (Figure S6E); while those of EPG were associated with “regulation of binding”, “protein localization to cell surface”, and “cellular response to cadmium ion” (Figure S6F). Notably, TRRUST analysis in Metascape indicated both DEGs of BPG and EPG were under the regulation of transcriptional factor *Stat3* (Figure S6E-F, bottom panel).

### DEHP impaired the interaction of female germ cells and pre-granulosa cells during PF formation

**Figure 6:**
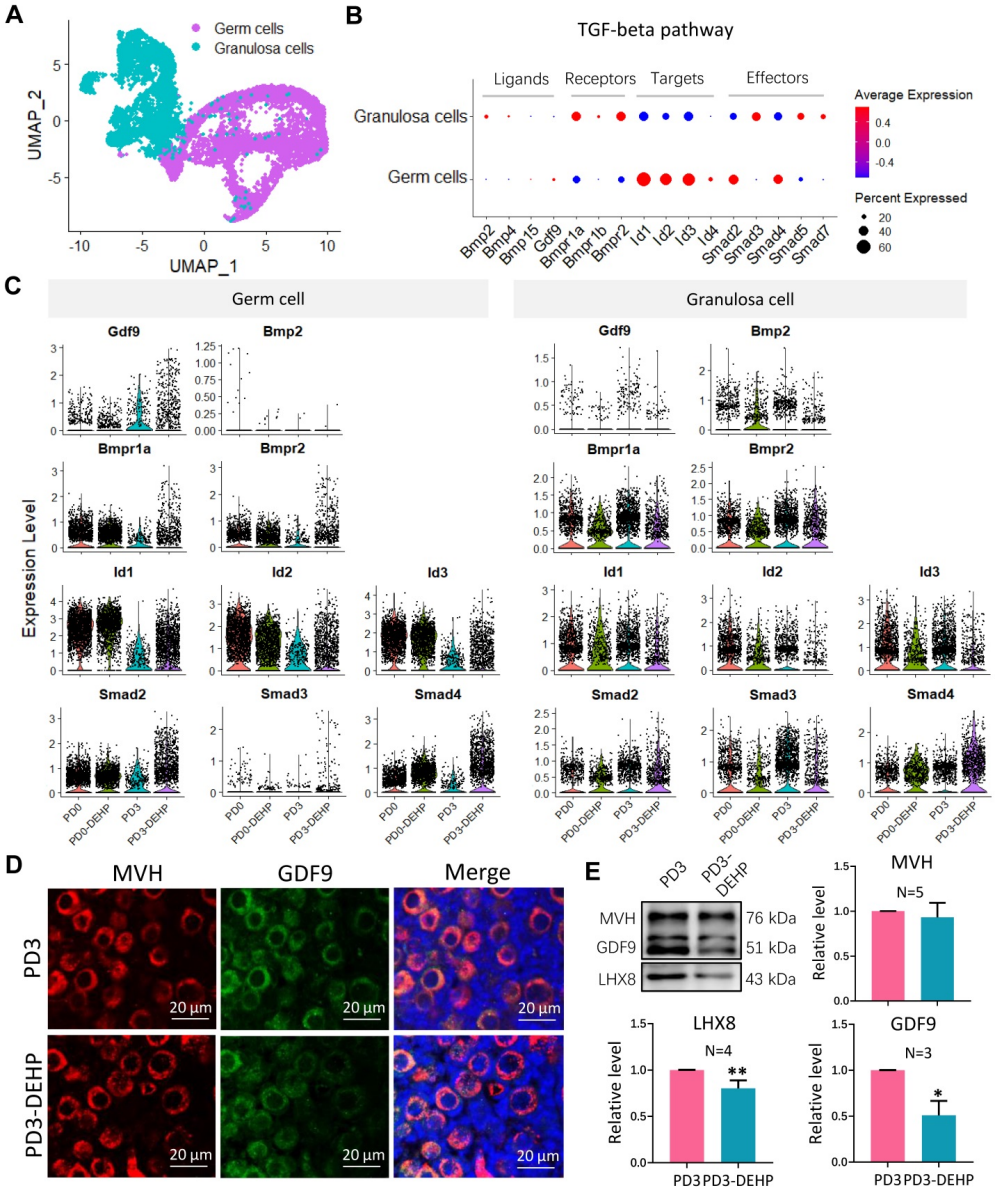
** DEHP devastated TGF-beta signal between germ cells and granulosa cells and further impaired follicular assembly. (A)** UMAP plot of separated germ cell and granulosa cell subpopulations. **(B)** Dot plot of components of TGF-beta signal (including signal factors, receptors, target genes, and effectors) between germ cells and granulosa cells. **(C)** Components of TGF-beta signal between sample groups in germ cells (left) and granulosa cells (right). **(D)** Histological section staining of MVH (red) and GDF9 (green) with and without DEHP treatment at PD3. Cell nuclei were stained with Hoechst 33342 (blue). Scale bar = 20 μm. **(E)** Detection of MVH, GDF9, and LHX8 protein levels at PD3 with and without DEHP exposure. MVH was used as a loading control to calculate relative protein levels of GDF9 and LHX8. Data are shown as mean ± SD (N represents independent repeats). * *P* < 0.05; **P < 0.01.

The two datasets of female germ cells and pre-granulosa cells during PF formation, were integrated to investigate the interaction (Figure 6A, and Figure S7A-B). Using the gene differential expression analysis above, the key DEGs affected by DEHP produced 194 genes in germ cells (DEGs detected by at least two strategies) and 138 genes in granulosa cells (Figure S7C-D). Enrichment of germ cells in the processes of “oxidative phosphorylation”, “cytoplasmic translation” and “cellular responses to stress”, and mTORC1 signaling were also included (Figure S7E). For granulosa cells, the terms of “ribosome assembly”, regulation of catabolic process and cell death, as well as WNT signaling and reactive oxygen species were also enriched (Figure S7F). It is noteworthy that the “Downregulation of SMAD2/3: SMAD4 transcriptional activity” term was enriched in both cell types. Thus, components of the TGF-beta pathway were highlighted in subsequent analysis (Figure 6B). In the results, most known elements regarding TGF-beta signaling were expressed by both or either of germ cells or granulosa cells. Results suggested that* Gdf9* and *Bmp2* were slightly expressed by germ cells and granulosa cells, respectively, while the receptors were mostly expressed by granulosa cells, and the target genes and effectors were highly expressed by germ cells. Moreover, the effect of DEHP on germ cells was not apparent, except for a reduction of *Gdf9* at PD3 and a rise in the levels of *Bmpr1a* and *Smad4* (Figure 6C, left). However, it caused upregulation of *Smad4* in granulosa cells, as well as a reduction of other genes such as the receptors of *Bmpr1a* and *Bmpr2*, targets of *Id2* and *Id3*, and effectors of *Smad2* and *Smad3* (Figure 6C, right)*.* Furthermore, the ovarian section staining of GDF9 protein revealed its location in germ cells (Figure 6D), Western blot in the protein level exhibited a decline of GDF9 and LHX8 after DEHP treatment (Figure 6E), and BMPR1A and SMAD3 expression was also affected by DEHP exposure (Figure S7G-H).

### DEHP leveraged apoptosis in both germ cells and somatic cells

**Figure 7:**
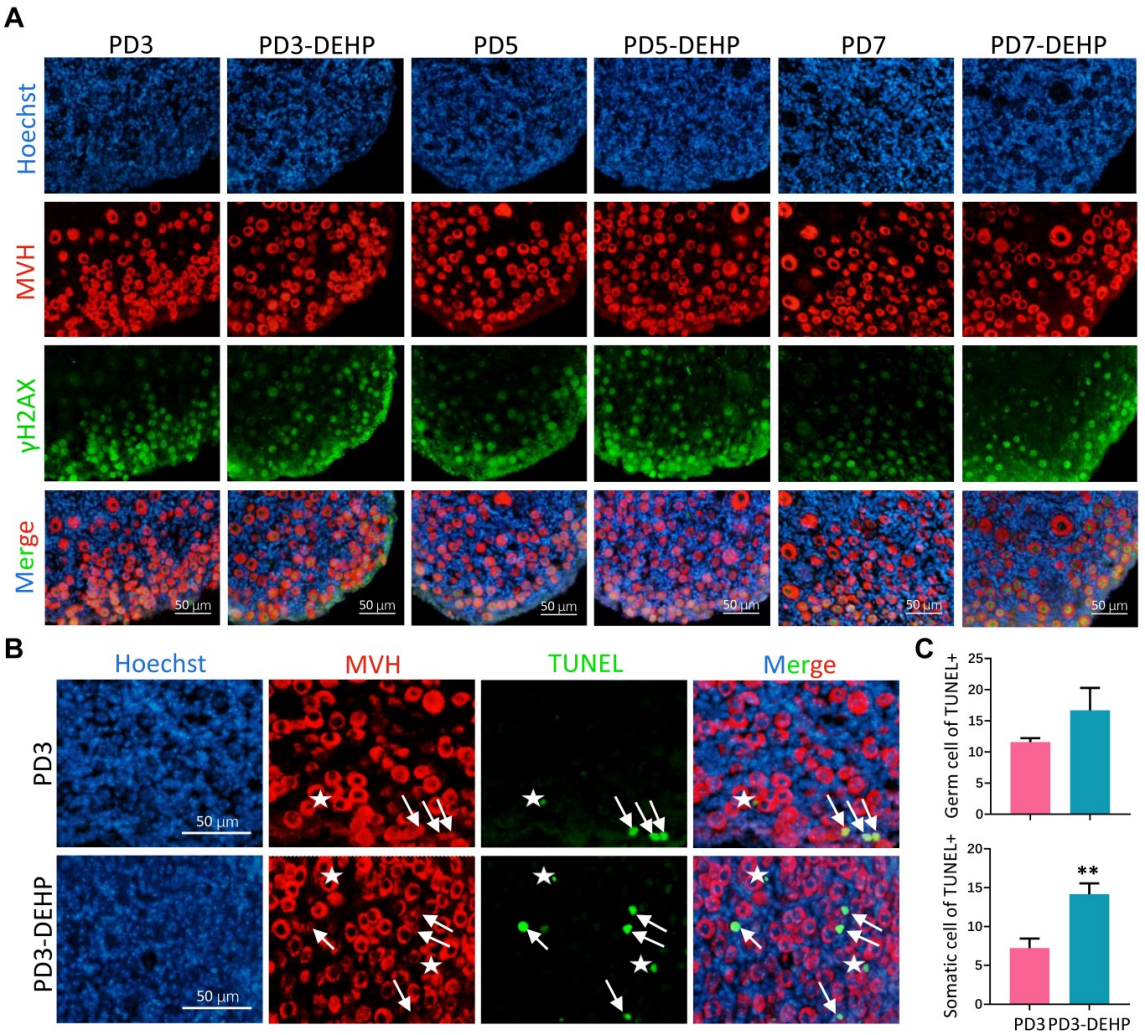
** DEHP triggered apoptosis in germ cells and ovarian somatic cells. (A)** Immunohistochemical staining of MVH (red) and γH2AX (green) in ovaries with and without DEHP treatment at PD3, PD5, and PD7. Cell nuclei were stained with Hoechst 33342 (blue). Scale bar = 50 μm. **(B)** TUNEL (green) and MVH (red) staining of ovarian sections at PD3. Cell nuclei were stained with Hoechst 33342 (blue). White arrows and pentagram mark the germ cells and granulosa cells with TUNEL, respectively. Scale bar = 50 μm. **(C)** The number of TUNEL positive germ cells (upper) and granulosa cells (lower) for each section at PD3. Data are shown as mean ± SD. **P < 0.01.

To uncover the destiny of germ cells in lagging cysts affected by DEHP, the DNA double strand break (DSB) and apoptotic processes were examined. The staining of ovarian sections showed that the double-positive of MVH and γH2AX (phosphorylated histone H2AX, served as a marker of DSB) in germ cells was raised by DEHP at PD3, as well as PD5 and PD7 (Figure 7A). In addition, the terminal deoxynucleotidyl transferase dUTP nick end labeling (TUNEL) assay detected that germ cells and ovarian somatic cells both underwent apoptosis (Figure 7B), and the numbers of TUNEL positive cells in these cell types increased (Figure 7C). These results suggested that DEHP exposure of pregnant mothers would trigger the apoptosis of pup ovarian cells.

### DEHP effects on immune cells and stromal cells during PF assembly

Further, the effects of DEHP on ovarian somatic cells were investigated. For immune cells, eight clusters were produced, and DEHP-treated cells at PD3 seemed to be more distinct from others (Figure S8A). Gene differential expression revealed that DEHP up- and down-regulated 10 genes respectively at PD0, and 26 genes were reduced but 88 genes were increased at PD3 (Figure S8B). GO terms of DEGs at PD0 enriched the biological processes of “multi-organism”, “immune system” and “localization”, while the top terms at PD3 were “immune system process”, “cell proliferation”, and “response to stimulus”. In terms of stromal cells, six clusters were generated, and the expression pattern of cells in the PD3-DEHP group was largely different from others (Figure S8D). Particularly, DEHP hardly affected stromal cells at PD0, with only one DEG (*Birc5*) being detected; at PD3, there were 12 down- and 75 up-regulated genes as a result of DEHP exposure (Figure S8E). Functional enrichment of DEGs at PD3 suggested the greatest enrichment of “cell proliferation”, “reproductive process”, and “cellular component organization or biogenesis” (Figure S8F).

## Discussion

DEHP is widely used as a plasticizer and is ubiquitous in every-day products; its toxicity has been well demonstrated by numerous studies 28, 51, including multiple human body systems, such as neural, endocrine, cardiotoxic, and reproductive 31, 52, 53. Meanwhile, during PF assembly in mammals, the multicellular regulatory networks make the parsing of its mechanism tough and many biological enigmas remain unknown. In the current study, using a maternal perinatal exposure of DEHP from 16.5 dpc to postnatal day 3, scRNA-seq was used to investigate the alteration of transcriptome programs in pup ovaries to explore how the gene regulation networks and cell interaction were affected by DEHP during PF formation in mice.

**Figure 8:**
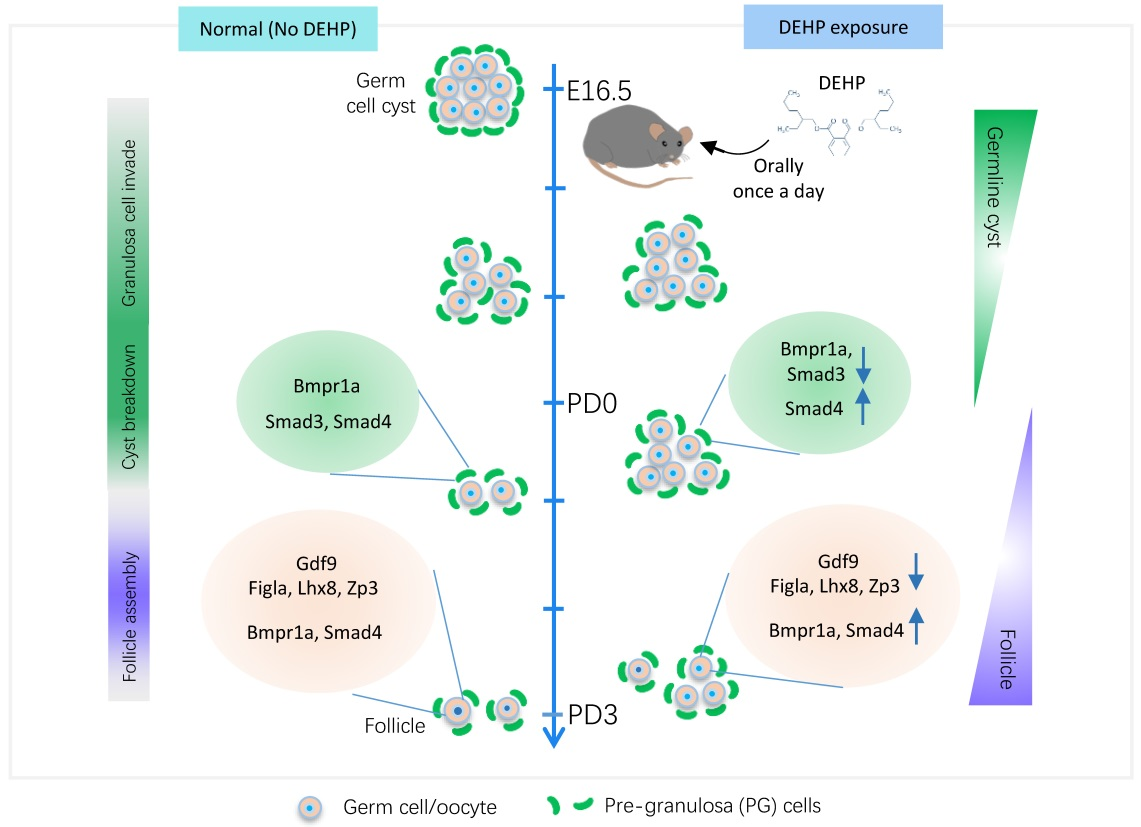
** The transcriptional dynamics of DEHP decaying primordial follicle formation.** The middle line marks the time window of primordial follicle formation, which represents germ cell fate transition from germline cysts to follicles (right annotation text), however, it consists of three main biological events, granulosa cell invasion, cyst breakdown, and follicle assembly (left annotation text). The left panel indicates normal follicle formation without DEHP, consisting of normal expression levels of *Figla*, *Lhx8,* and *Zp3,* and TGF-beta members (*Gdf9*, *Bmpr1a,* and *Smad4*) in germ cells, as well as TGF-beta receptors (*Bmpr1a*) and effectors (*Smad3* and *Smad4*) in pre-granulosa cells. The right panel displays follicle assembly impaired by DEHP, which shows the decreasing transcripts of key genes for follicle formation (*Figla*, *Lhx8*, and *Zp3*) and of TGF-beta components in germ cells (*Gdf9*) and pre-granulosa cells (*Bmpr1a* and *Smad3*), but the increasing levels of *Smad4* in both cell types and *Bmpr1a* only in germ cells.

Great progress has been made since single-cell transcriptomics has been applied to studies of the female reproductive system 43, 45, 49, 50. Specifically, the transcriptome landscape of the whole process of folliculogenesis in humans has been described, and single-cell transcriptomic analyses of monkey ovaries have identified ovarian aging associated dysregulation of antioxidative pathways 54. The current study identified six types of ovarian cells, which was similar to previous scRNA-seq reports using the same timeline in murine ovaries 49, 50. Subsequently, the biological toxicity of DEHP on germ cells was highlighted. Firstly, from a single-cell perspective, DEHP decreased the expression levels of genes essential for PF assembly (Figure 8, right panel for germ cell), especially at PD3, such as *Figla* and *Lhx8*. Similarly, it is reported that DEHP exposure also decreased the levels of oocyte-specific genes, such as *Lhx8, Figla, Sohlh1,* and *Nobox* 25. Furthermore, DEHP inhibited germline cyst breakdown and impaired PF assembly 26, 55, 56. Nevertheless, the current study displayed an impeded progression of PF assembly through a decline of the cell percentages of early-stage follicles in PD0-DEHP and late-stage in the PD3-DEHP group. Further, the ovarian sections revealed that DEHP treatment significantly reduced the cell percentage of germ cells within follicles.

Moreover, the process of cellular response to stress and DNA damage stimulus, as well as oxidative phosphorylation and transcriptional regulation by TP53, were all enriched after DEHP exposure. All these changes seemed to be associated with cellular stress, because TP53 (also termed tumor protein p53) is a DNA sequence-specific transcriptional regulator responsive to various forms of cellular stress and is involved in cell cycle arrest and apoptosis 57, 58. Moreover, oxidative phosphorylation is the main source of reactive oxygen species (ROS) in mitochondria 59. Oxidative phosphorylation and mitochondrial activity were also observed in pseudotime analysis, which were largely affected by DEHP. The *in vitro* study demonstrated that the oxidative stress-related gene *Xdh* (xanthine dehydrogenase) may serve as the downstream target of DEHP, whose reduction (through *Xdh* RNAi or melatonin supplement) can inhibit the increasing levels of ROS and mitigate the damage to PF assembly caused by DEHP 55. Thus, DEHP, as one EDC member, might impair PF assembly through oxidative stress. In terms of molecular mechanisms, previous research suggests that ERs, particularly ER-beta, participate in the DEHP-mediated effect on ovarian tissue 26, 55. In addition, the alteration of DNA methylation and transgenerational actions by EDCs in germ cells has been well documented 8, 24, 60-62. In fact, H3K4 mono-methylation was likely affected by DEHP in the present study (Figure 4E). A recent study of* Caenorhabditis elegans* reports that DEHP exposure alters the expression of the reproduction-related gene *spr-5*, which encodes H3K4me2 demethylase 63; however, a similar effect of DEHP on H3K4 mono-methylation in the mouse still needs to be studied.

The effect of DEHP on PG cells and its interaction with germ cells during PF formation was also explored. It is known that DEHP impairs the interaction of germ cells and PG cells via decreasing NOTCH2 signaling and impeding the proliferation of PG cells during PF assembly 26, which seemed to be evidenced, at least partially, by the enriched process of the regulation of cyclin-dependent protein kinase activity in the current study, as well as a positive regulation of the cell cycle in BPG. In addition, the death of both EPG and BPG cells was potentially affected by DEHP (Figure 5G-H). Previous research shows that DEHP exposure increases apoptosis of granulosa cells in adults, which leads to an increase in the number of atretic follicles 64, 65. Similarly, during the perinatal period, TUNEL tests previously suggested that DEHP exposure *in vitro* increases apoptosis in oocytes of neonatal ovaries 25, which is consistent with this study (Figure 7B). Meanwhile, the components of the TGF-beta pathway in germ cells and PG cells were disturbed by DEHP (Figure 8, right panel for PG cells). For instance, *Gdf9* on germ cells, and BMP receptors (*Bmpr1a* and* Bmpr2*) on PG cells, as well as targets (*Id2* and* Id3* on germ cells) and effectors (*Smd2, Smd3* and* Smad4* both on germ cells and PG cells), were all influenced by DEHP exposure (Figure 6B-C). Furthermore, GDF9 protein level was more lowly expressed, whilst BMPR1A and SMAD3 were up more highly expressed following DEHP exposure. Growing evidence indicates that TGF-beta signaling is important in the regulation of cyst breakdown and PF assembly 14, 15, 66. Despite some available connections between DEHP and TGF-beta signaling 67, 68, DEHP toxicity mediated by TGF-beta signaling is still poorly understood, and needs to be further explored.

In conclusion, using scRNA-seq, the current study investigated the transcriptional dynamics of germ cells and PG cells following prenatal DEHP exposure during PF assembly. The results highlighted that DEHP impaired germline cyst breakdown and PF formation might be caused by oxidative stress in germ cells. Moreover, DNA damage and apoptosis were increased, as well as TGF-beta signaling being disturbed between both cell types. The current research presents a systematic understanding of DEHP damage on murine PF formation, and offers a preventive and therapeutic schedule for its biotoxicity in humans.

## Materials and methods

### Animals, reagents, and preparation for cell suspension

C57BL/6J strain mice, used only for single-cell sequencing, were purchased from Vital River Laboratory Animal Technology Co. Ltd (Beijing, China). For biochemical experiments, CD-1 mice were purchased from Jinan Pengyue Experimental Animal Breeding Co. Ltd (Jinan, China). The mice were raised in cages with *ad-libitum* food and water, a 12-h light:12-h dark cycle at a constant temperature (24 ℃) and humidity. Females were mated with reproductive males around 17:00 h. The following morning, a vaginal plug indicated a successful mating, and the time was recorded as 0.5 dpc. At 16.5 dpc, mice were treated with 40 μg/kg body weight DEHP via oral gavage once a day. Postnatal female pups (birth on 19.5 dpc) at 0 and 3 days (PD0 and PD3) were sacrificed for subsequent isolation of ovarian tissue. In accordance with national guidelines, all the murine experimental procedures were approved by the Animal Care and Ethical Committee of Qingdao Agricultural University.

For humans, the estimated DEHP dose of daily exposure ranges from 3 to 30 μg/kg body weight 7. In the current study, DEHP was purchased from Sigma (Sigma-Aldrich, 36735-1G, Shanghai, China), the stock solution of which was prepared in DMSO. Before oral gavage, the working solution was diluted with sterile water. Regarding DEHP dose, 40 μg/kg body weight daily was adopted in this study, following reference to many *in vivo* publications 21, 51, 69 and previous papers 30, 34, 35.

For the preparation of single cells, the ovarian tissues (4-6 ovaries per sample) were cut into pieces within the digestive solution, which comprised 0.25% trypsin (Hyclone, Beijing, China) and collagenase (2 mg/ml, Sigma-Aldrich, C5138). Subsequently, the mixture was transferred into centrifuge tubes and placed in a 37 ℃ incubator for 6-8 min, and then agitated with a pipette once every 3 min to produce single cells. After the termination of digestion with serum, the mixture was then filtered using 40 μm cell strainers (BD Falcon, 352340, CA, USA), and flushes were performed three times with PBS containing 0.04% BSA. Finally, cell viability and counts were assessed to meet the requirements of sequencing.

### Single-cell libraries and sequencing

The single cells were then loaded onto a 10× Chromium chip B (10× Genomics, Pleasanton, CA, USA) to generate single-cell gel beads in emulsion (GEMs), which was followed by cDNA amplification. A 3ʹ gene expression library was then constructed using a 10× Genomics Chromium Single Cell system with v3 chemistry (10× Genomics) according to the manufacturer's instructions. Sequencing was performed on a NovaSeq 6000 (Illumina) by Berry Genomics (Chengdu, China). Output reads (150 bp pair-ended) were yielded through Cell Ranger v3.1.5 using default parameters, including “mkfastq” and “count” pipelines. The detailed information of captured cells for each sample after “count” flow is shown in Figure S1B.

### Data analysis using the Seurat and Monocle package

R package “Seurat” v3.1.2 was used for data pre-processing, and parameters were set and adjusted according to the features of captured transcripts in each sample. Doublets were examined by DoubletFinder v2.0.3 with its default threshold value of 0.075 70. Meanwhile, a double-check for every type was executed to discard cells with abnormal gene expression (for example, cells that were over-high in a number of unique detected genes, or cells within a single-cell type but with highly expressed specific marker genes in other cell types). The four objects were integrated via “Anchors” (an integration strategy between single-cell datasets for identification of cell pairwise correspondences and constructing atlases into their shared space) 71; through normalization, datasets scaling, and test for optimal parameter combinations of “dim.use” and “resolution”, clustering was generated with a series commands of “FindNeighbors”, “FindClusters”, and “RunUMAP”. In addition, the “FindAllMarkers” function was applied to identify marker genes in clusters, while gene differential expression analysis was performed with default parameters; the significantly expressed genes (DEGs) were defined with |avg_logFC| > 0.25 and “p_val_adj” < 0.01.

The Monocle package (v2.10) 72 was used to construct a single-cell pseudotime trajectory using the functions of “reduceDimension” and “orderCells”. Ordering genes were selected based on highly variable genes identified by Seurat. In particular, “State” is Monocle's jargon for the segment of the trajectory tree in pesudotime analysis. Moreover, the “BEAM” function was used to calculate the dynamic genes at the branch point, and the genes with “qval < 1e-4” were displayed using heatmaps.

### Functional enrichment and mapping

The online tool, Metascape 73 was used to perform the enrichment analyses of gene ontology (GO), and Kyoto encyclopedia and genomes (KEGG), including protein-protein interaction and transcriptional factor of TRRUST. EnrichmentMap was established using the g.profiler (https://biit.cs.ut.ee/gprofiler/gost) with the gene list as input and results were visualized in Cytoscape (v3.7.2) with cutoff at 0.01 of “FDR q-value” 74.

### Histological section staining

Ovaries were collected and fixed in 4% paraformaldehyde (Solarbio, P1110, Beijing, China) overnight. Ovaries were embedded in paraffin and 5 µm sections were prepared. Following a standard procedure of gradient rehydration and antigen retrieval, sections were blocked at a room temperature around 20 ℃ for 45 min, then primary antibody incubation was performed overnight at 4 ℃. The primary antibodies included rabbit polyclonal to MVH (Abcam, ab13840, Shanghai, China), AMHR2 (OriGene, TA323994, Rockville, USA), GDF9 (Abcam, ab93892), and mouse monoclonal to γH2AX (Abcam, ab26350), STAT3 (Cell Signaling Technology, #91395, Danvers, USA), and MVH (Abcam, ab27591). The next day, the secondary antibodies (Abcam, Goat Anti-Rabbit IgG H&L Alexa Fluor 488, ab150077, and Anti-Mouse, ab150113; Donkey Anti-Rabbit IgG H&L Alexa Fluor 555, ab150074, and Anti-Mouse, ab150106) were incubated at 37 ℃ for 30 min. After nuclei staining with propidium iodide (PI, Sigma-Aldrich, P4170) or Hoechst 33342 (Beyotime, C1022, Shanghai, China), each slide was sealed with anti-fluorescence attenuation solution (Boster, AR1109, Wuhan, China). Photographs were then taken with a LEICA TCS SP5 II confocal microscope (Leica, Wetzlar, Germany). In addition, a single-germ cell without connection to other germ cells was considered as a germ cell in a follicle, while inter-connected germ cells including two or more in number were regarded as germ cells in cysts. Meanwhile, every one of five ovarian sections was counted, and a total of eight to ten sections for each ovary was viewed as one independent biological duplicate.

### TUNEL assay

The stained ovarian sections were examined using a TUNEL BrightGreen Apoptosis Detection Kit (Vazyme, A112-03, Nanjing, China). According to the instructions for usage, after incubation of the secondary antibody for MVH in histological staining workflow, the slides were washed twice with PBS and treated with proteinase K for 10 min. Following two further washes with PBS, the slides were incubated with 1 × Equilibration Buffer for 25 min at room temperature, and the BrightGreen Labeling Mix was applied at 37 ℃ for 80 min. Then, the samples were counterstained with Hoechst 33342 for cell nuclei and sealed for observation under a fluorescence microscope (Olympus, BX51, Japan).

### Western blot

Reference was made to previously developed protocols 55, 75; proteins from six ovaries were extracted with RIPA lysis solution (Beyotime, P0013C). After denaturation treatment, the protein of each sample was separated by SDS-polyacrylamide gel electrophoresis (SDS-PAGE) and transferred onto a polyvinylidene fluoride (PVDF, Millipore, ISEQ00010, USA) membrane. Subsequently, protein bands were blocked at a temperature of approximately 20 ℃ for 2 h, and the primary antibody was incubated at 4 ℃ overnight. The following day, after three washes, the secondary antibodies were incubated. Finally, a BeyoECL Plus kit (Beyotime, P0018) was used for chemiluminescence. The primary antibodies used for Western blot consisted of MVH (Abcam, ab13840), GDF9 (Abcam, ab93892), LHX8 (Abcam, ab137036), BMPR1A (Affinity, DF6634, Shanghai, China), SMAD3 (Abcam, ab40854), and GAPDH (Affinity, AF7021).

### Statistical analysis

Results are shown as mean ± SD from at least three independent experiments. Statistical analyses were performed with GraphPad Prism software (version 8.0). Significant differences were determined using an unpaired t-test; significant and highly significant differences were defined at * *P < 0.05* and ** *P < 0.01*, respectively.

## Supplementary Material

Supplementary figures.

Supplementary table 1: Top-50 marker genes of cell clusters in ovaries.

Supplementary table 2: Top-50 marker genes of germ cells.

Supplementary table 3: Result of differential gene expression analysis at pre-stage of follicle formation in germ cells.

Supplementary table 4: Result of differential gene expression analysis at early-stage of follicle formation in germ cells.

Supplementary table 5: Result of differential gene expression analysis at late-stage of follicle formation in germ cells.

Supplementary table 6: Result of differential gene expression analysis in BPG cells.

Supplementary table 7: Result of differential gene expression analysis in EPG cells.
