# Supplementary material for: Single-cell transcriptome dissection of the toxic impact of Di (2-ethylhexyl) phthalate on primordial follicle assembly
Source: Theranostics. 2021 Mar 5;11(10):4992–5009. doi: 10.7150/thno.55006 (PMC7978297; doi:10.7150/thno.55006)
Supplement: Supplementary file 1 — Supplementary figures. [file thnov11p4992s1.pdf]

Supplemental Figures

Fig S1. Detailed dataset for pre-processing and cell types identified in ovaries

(A) UMAP plot of single and doublet detection in four datasets. (B) The detailed information for sample pre-processing parameters during CellRanger and Seurat workflow. (C) Heatmap of the top five marker genes identified in each cell cluster in ovaries. (D) Violin plots of representative genes for germ cells, granulosa cells, stromal cells, erythrocytes, immune cells, and endothelial cells.

Fig S2. Histological section staining of germ cells and granulosa cells

(A) Histological section staining of STAT3 (green) for germ cells in ovaries with and without DEHP treatment at PD3. Cell nuclei were stained with PI (red). (B) Histological section staining of AMHR2 (green) for granulosa cells in ovaries with and without DEHP treatment at PD3. Cell nuclei were stained with Hoechst 33342 (blue). Scale bar = 50 μm.

Fig S3. Single-cell analysis of germ cell population following DEHP exposure

(A) Cell-cycle-phase score of germ cell population using the gene list adopted from Tirosh et al. [76]. (B) Heatmap of the newly identified top five marker genes in germ cell clusters. (C) Feature plots of key genes with regard to meiosis, follicle formation, and oocyte growth in germ cell clusters. (D) DEHP down-regulated the expression of genes critical to follicle assembly. (E) Pathways and processes enriched by 14 key DEGs at the pre-stage of PF formation.

Fig S4. Stage-specific DEG enrichment in germ cells

(A) Enrichment of DEG lists with PD0-DEHP *vs.* PD0 and PD3-DEHP *vs.* PD3 at the pre-stage of PF formation in germ cells. (B) Enrichment of DEG lists with PD0-DEHP *vs.* PD0 and PD3-DEHP *vs.* PD3 at the early-stage of PF formation in germ cells. (C) Enrichment of DEG lists with PD0-DEHP *vs.* PD0 and PD3-DEHP *vs.* PD3 at the late-stage of PF formation in germ

cells.

Fig S5. Single-cell pseudotime in germ cell trajectories

(A) The representative genes mark different developmental States in germ cell trajectories. (B) Expression of representative genes in each gene set regarding fate transition in germ cells colored by cell States. (C) Enrichment of gene set 4 for protein-protein interaction by Metascape. (D) Heatmap of two gene sets between different fates of germ cells at branch point two. Several representative genes of each gene set are listed in the right panel. (E) Expression plots of meiotic (*Smc1b, Taf7l*) and oocyte-specific (*Ooep, Padi6*) genes along pseudotime.

Fig S6. Differential expression of two groups of pre-granulosa cells

(A) Feature plots of marker gene expression in BPGs based on UMAP. (B) Feature plots of marker gene expression in EPGs based on UMAP. (C) Venn diagram of DEGs at PD0 and PD3 in BPGs. (D) Venn diagram of DEGs for PD0 and PD3 in EPGs. (E) Enrichment analysis of 192 key genes in BPGs for protein-protein interaction (upper) and transcriptional factors of TRRUST (below) in Metascape. (F) Enrichment analysis of 248 key genes in EPGs for protein-protein interaction (upper) and transcriptional factors of TRRUST (below) in Metascape.

Fig S7. Enrichment analysis of key DEGs both in germ cells and pre-granulosa cells

(A) UMAP plot of germ cell and granulosa cell populations colored by sample groups. (B) UMAP feature plots of marker genes for germ cells (left) and granulosa cells (right). (C) Venn diagram of DEGs in germ cells for pre-, early-, and late-stages of follicle formation and State in Monocle. Key DEGs are marked with a dashed red line. (D) Venn diagram of DEGs of BPGs and EPGs. (E) Enrichment terms of 194 key genes affected by DEHP in germ cells. (F) Enrichment terms of 138 key genes affected by DEHP in pre-granulosa cells. (G) RNA expression levels of *Bmpr1a* and *Smad3* in all ovarian cells with and without DEHP exposure. (H)

Detection of BMPR1A and SMAD3 protein levels in PD3 mouse ovaries with and without DEHP exposure. GAPDH as a loading control for relative protein levels of BMPR1A and SMAD3. Data are shown as mean ± SD (N represents independent repeats). **$P < 0.01$.

Fig S8. Differential expression of immune cells and stromal cells

(A) UMAP plot of immune cell populations colored by sample groups (upper) and clusters (below). (B) Volcano plots of gene differential expression in immune cells for PD0-DEHP *vs.* PD0 and PD3-DEHP *vs.* PD3. (C) Top GO terms of DEGs in immune cells at PD0 and PD3. (D) UMAP plot of stromal cells colored by sample groups (upper) and clusters (below). (E) Volcano plots of gene differential expression in stromal cells for PD0-DEHP *vs.* PD0 and PD3-DEHP *vs.* PD3. (F) Top GO terms of DEGs in stromal cells at PD3.

Figure S1

A

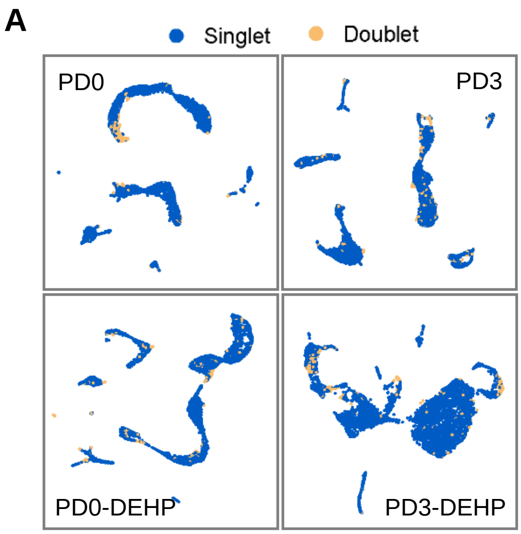

B

Category	PD0	PD0-DEHP	PD3	PD3-DEHP
Estimated Number of Cells	6,000	6,000	6,000	6,000
Valid Barcodes	97.4%	97.9%	97.5%	97.2%
Reads Mapped to Genome	92.4%	95.1%	93.4%	95.0%
Median Genes per Cell	2,844	2,720	2,768	1,164
Mean Reads per Cell	101,135	97,901	93,851	85,133
Sequencing Saturation	77.3%	58.1%	79.5%	70.5%
Threshold of nFeature RNA	> 1500 & < 7500	> 500 & < 9500	> 1500 & < 7500	> 200 & < 10000
Cell number after quality control	4270	4558	5375	5252
High-quality germ cell	1961	1418	308	2731
High-quality granulosa cell	1433	543	2383	926

C D

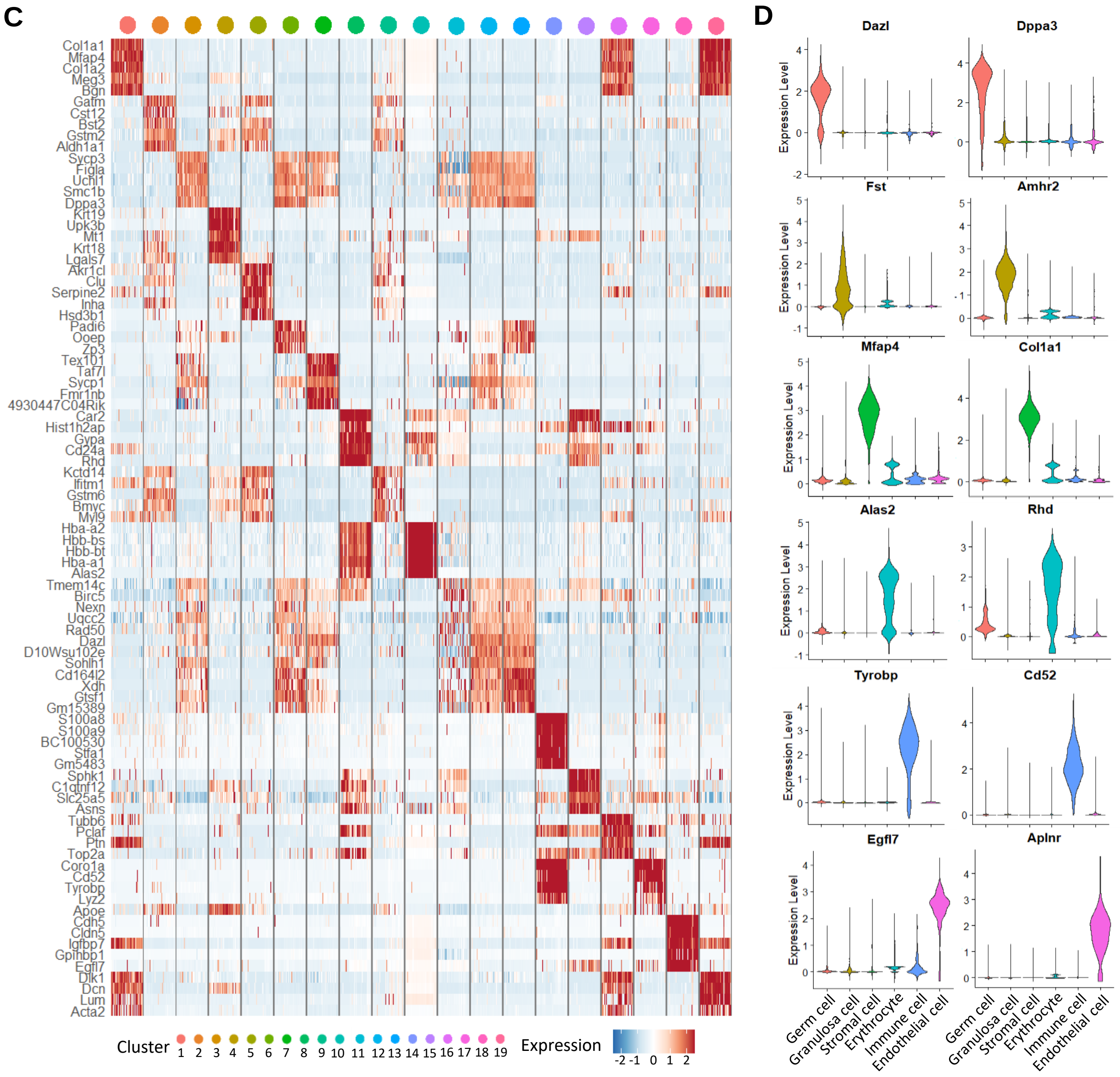

Figure S2

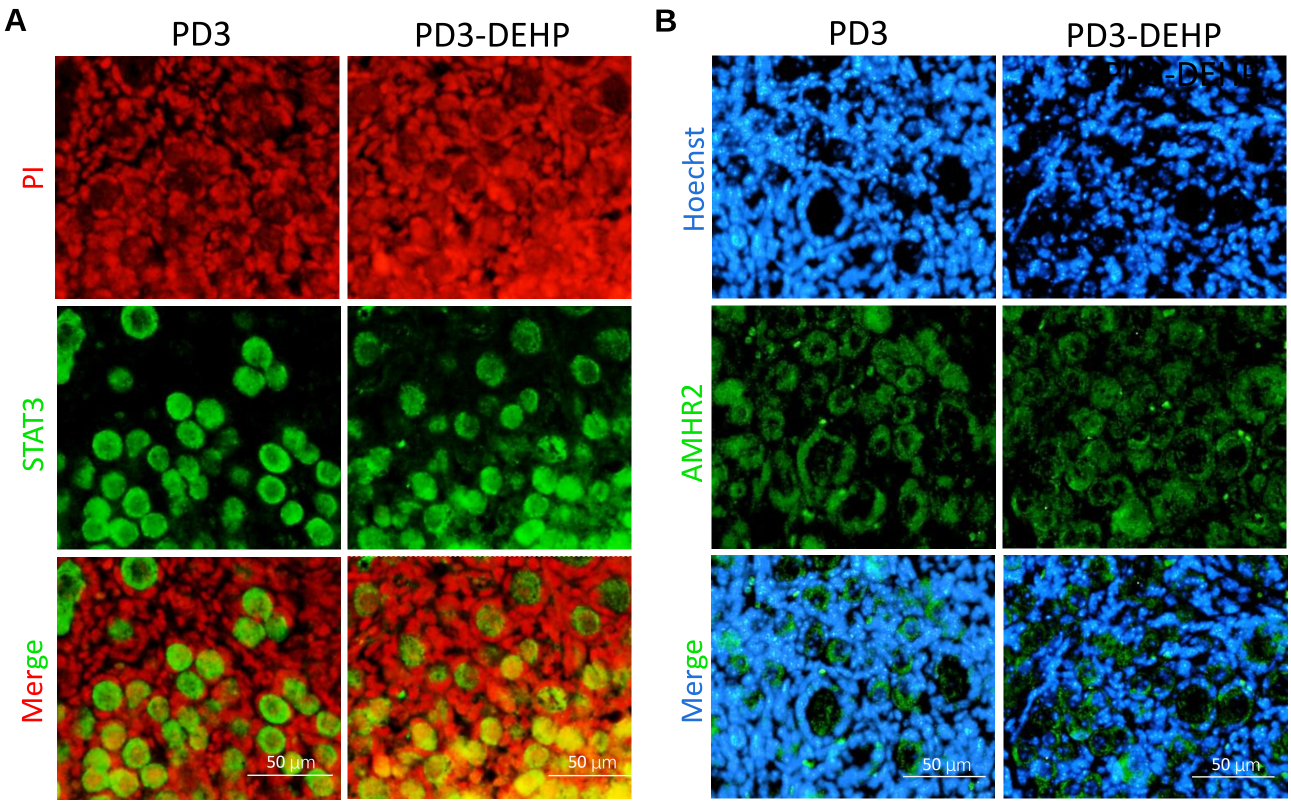
A
PD3
PD3-DEHP
PI
STAT3
Merge
50 µm
50 µm
B
PD3
PD3-DEHP
Hoechst
AMHR2
Merge
50 µm
50 µm

Figure S3

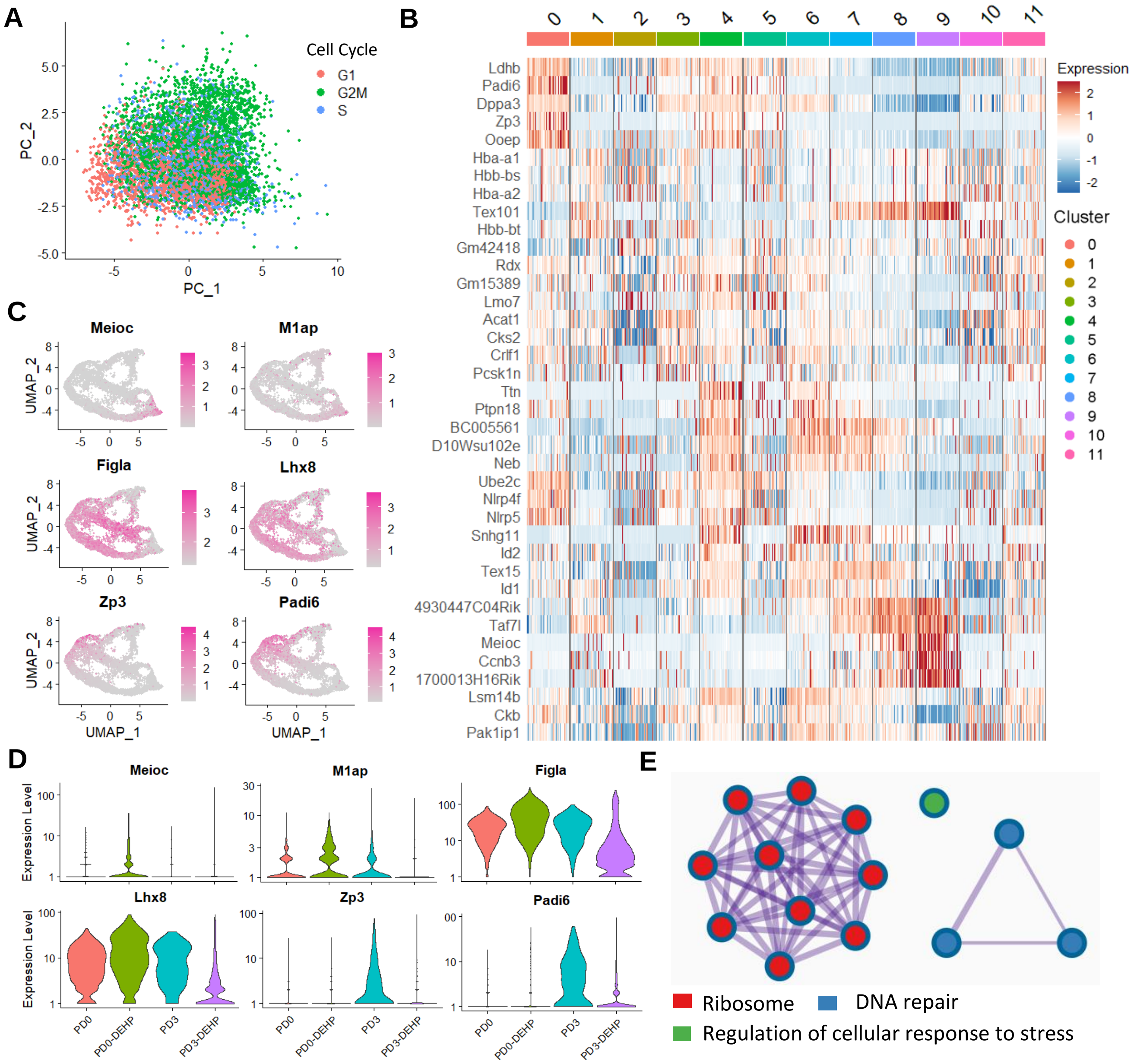
A
Cell Cycle
G1
G2M
S
PC_1
PC_2
B
Expression
Cluster
Ldhb
Padi6
Dppa3
Zp3
Ooep
Hba-a1
Hbb-bs
Hba-a2
Tex101
Hbb-bt
Gm42418
Rdx
Gm15389
Lmo7
Acat1
Cks2
Crlf1
Pcsk1n
Ttn
Ptpn18
BC005561
D10Wsu102e
Neb
Ube2c
Nlrp4f
Nlrp5
Snhg11
Id2
Tex15
Id1
4930447C04Rik
Taf7l
Meioc
Ccnb3
1700013H16Rik
Lsm14b
Ckb
Pak1ip1
C
Meioc
M1ap
Figla
Lhx8
Zp3
Padi6
UMAP_1
UMAP_2
D
Meioc
M1ap
Figla
Lhx8
Zp3
Padi6
Expression Level
PD0
PD0-DEHP
PD3
PD3-DEHP
E
Ribosome
DNA repair
Regulation of cellular response to stress

Figure S4

A

Enrichment analysis of DEG lists at pre-stage

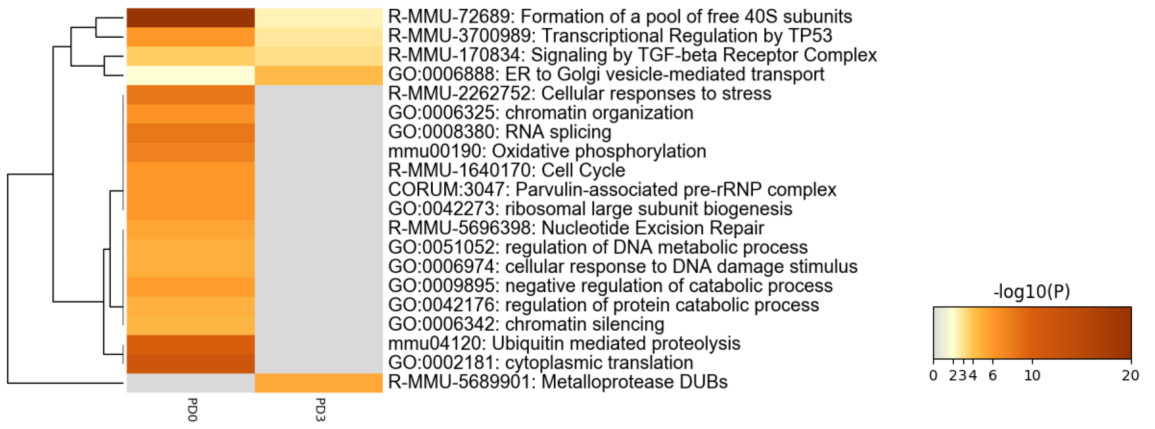

B

Enrichment analysis of DEG lists at early-stage

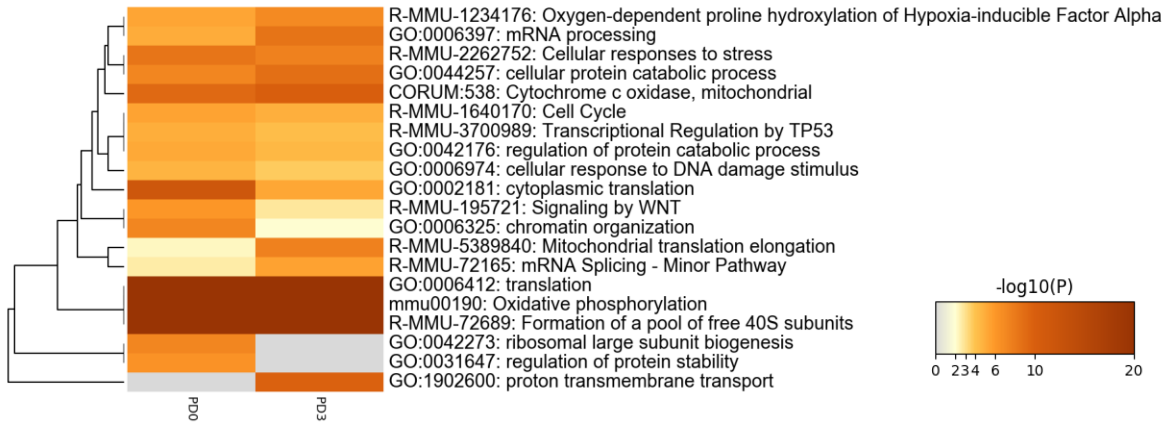

C

Enrichment analysis of DEG lists at late-stage

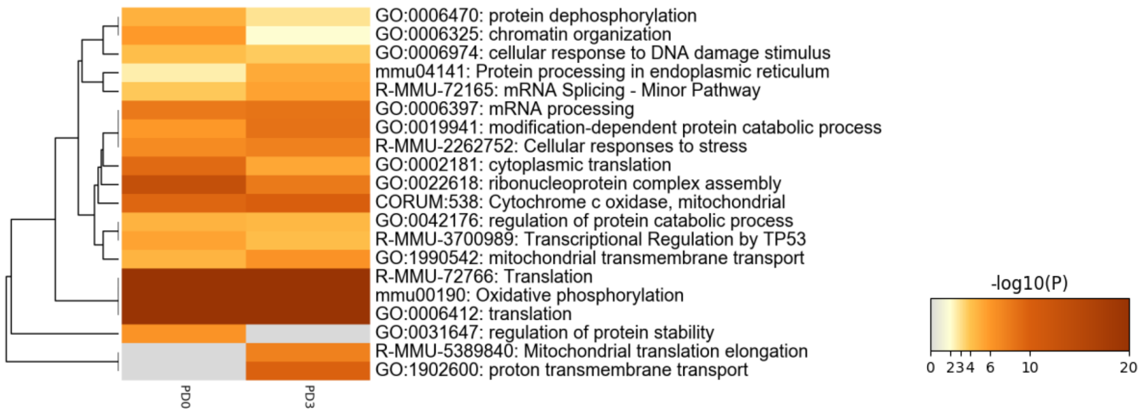

Figure S5

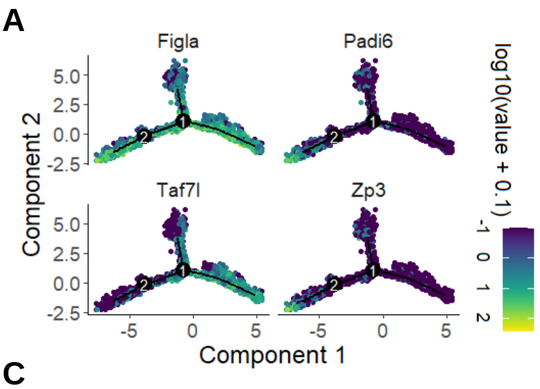

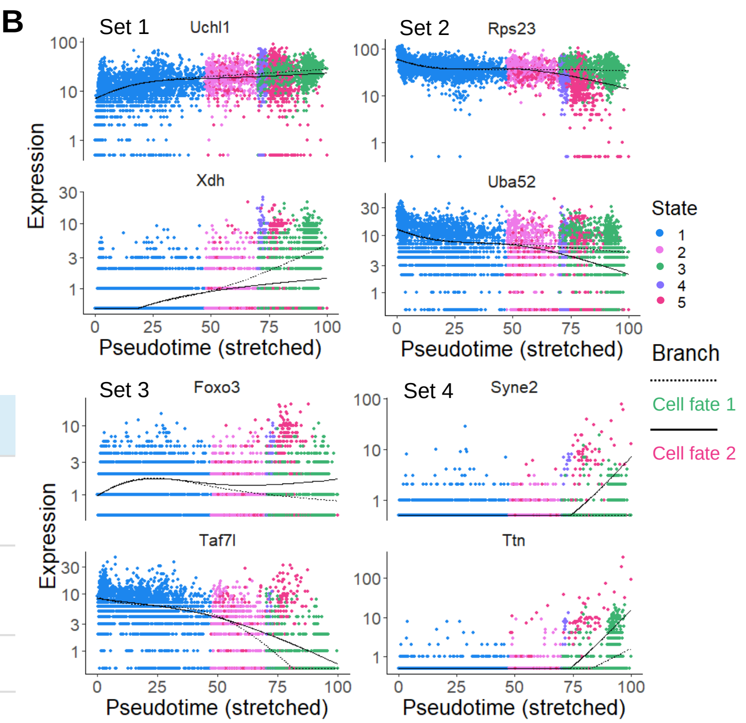

C

GO	Description	Log10(P)
GO:0006325	chromatin organization	-17.3
GO:0016569	covalent chromatin modification	-16.5
GO:0016570	histone modification	-15.9

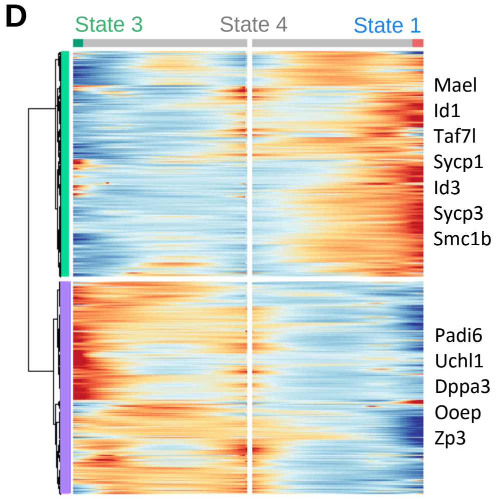

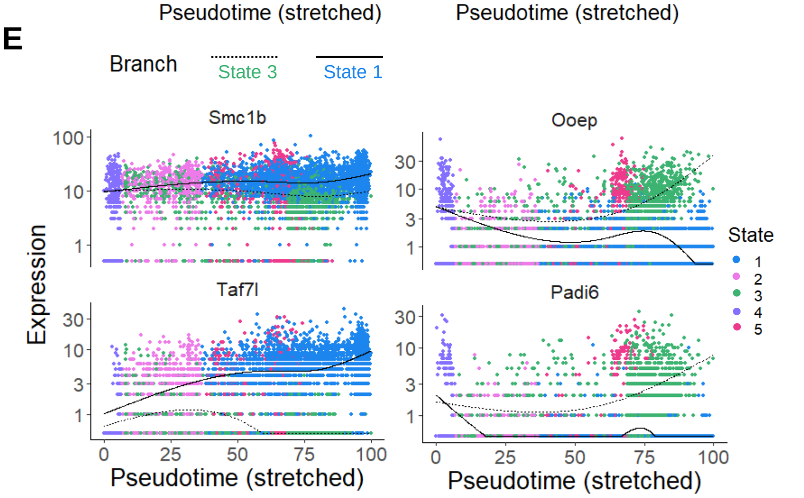

Figure S6

A

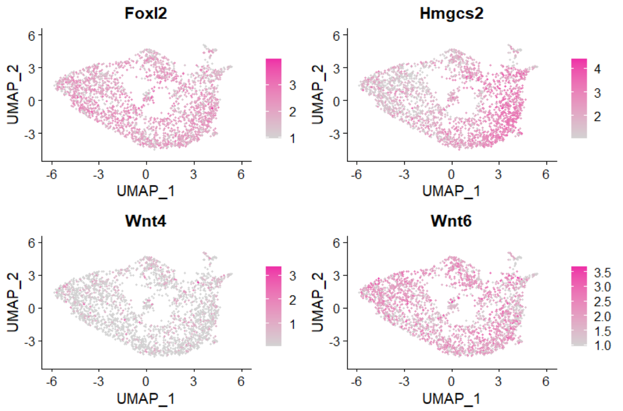

B

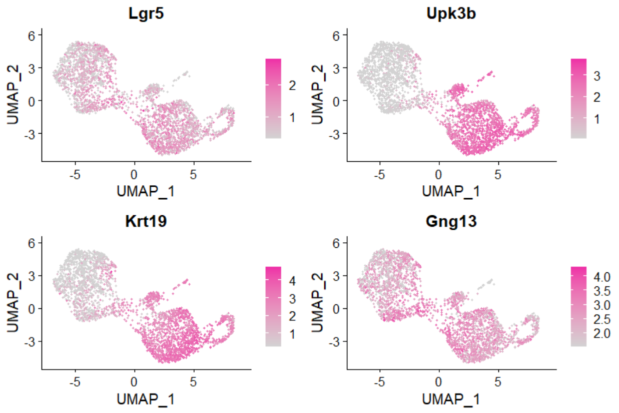

C

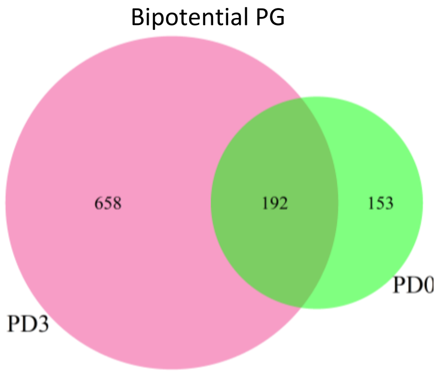

D

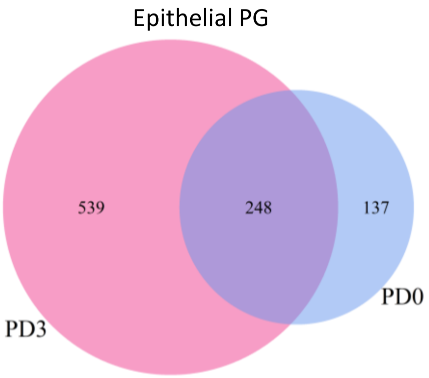

E

Protein-protein interaction enrichment analysis (192 genes)

GO	Description	Log10(P)
GO:1904031	positive regulation of cyclin-dependent protein kinase activity	-10.2
GO:1904029	regulation of cyclin-dependent protein kinase activity	-8.5
GO:0045787	positive regulation of cell cycle	-8.0

Summary of enrichment analysis in TRRUST.

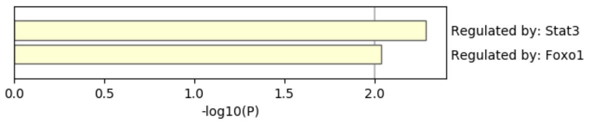

F

Protein-protein interaction enrichment analysis (248 genes)

GO	Description	Log10(P)
GO:0051098	regulation of binding	-6.2
GO:2000010	positive regulation of protein localization to cell surface	-6.1
GO:0071276	cellular response to cadmium ion	-6.1

Summary of enrichment analysis in TRRUST.

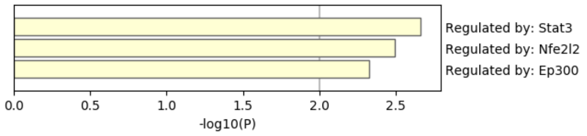

Figure S7

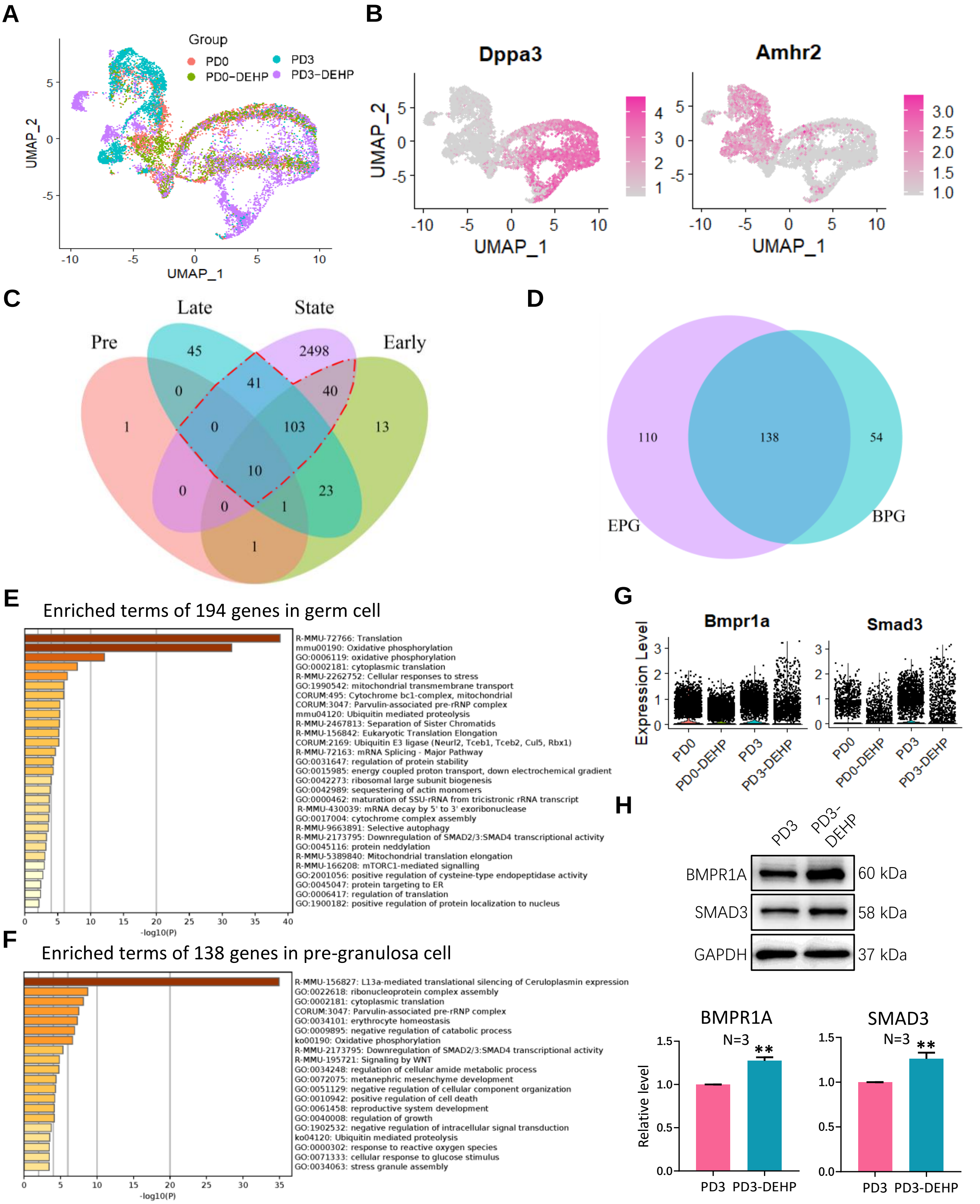

A
Group
PD0
PD0−DEHP
PD3
PD3−DEHP
UMAP_1
UMAP_2
B
Dppa3
Amhr2
UMAP_1
UMAP_2
C
Pre
Late
State
Early
D
EPG
BPG
110
138
54
E
Enriched terms of 194 genes in germ cell
R-MMU-72766: Translation
mmu00190: Oxidative phosphorylation
GO:0006119: oxidative phosphorylation
GO:0002181: cytoplasmic translation
R-MMU-2262752: Cellular responses to stress
GO:1990542: mitochondrial transmembrane transport
CORUM:495: Cytochrome bc1-complex, mitochondrial
CORUM:3047: Parvulin-associated pre-rRNP complex
mmu04120: Ubiquitin mediated proteolysis
R-MMU-2467813: Separation of Sister Chromatids
R-MMU-156842: Eukaryotic Translation Elongation
CORUM:2169: Ubiquitin E3 ligase (Neurl2, Tceb1, Tceb2, Cul5, Rbx1)
R-MMU-72163: mRNA Splicing - Major Pathway
GO:0031647: regulation of protein stability
GO:0015985: energy coupled proton transport, down electrochemical gradient
GO:0042273: ribosomal large subunit biogenesis
GO:0042989: sequestering of actin monomers
GO:0000462: maturation of SSU-rRNA from tricistronic rRNA transcript
R-MMU-430039: mRNA decay by 5' to 3' exoribonuclease
GO:0017004: cytochrome complex assembly
R-MMU-9663891: Selective autophagy
R-MMU-2173795: Downregulation of SMAD2/3:SMAD4 transcriptional activity
GO:0045116: protein neddylation
R-MMU-5389840: Mitochondrial translation elongation
R-MMU-166208: mTORC1-mediated signalling
GO:2001056: positive regulation of cysteine-type endopeptidase activity
GO:0045047: protein targeting to ER
GO:0006417: regulation of translation
GO:1900182: positive regulation of protein localization to nucleus
-log10(P)
F
Enriched terms of 138 genes in pre-granulosa cell
R-MMU-156827: L13a-mediated translational silencing of Ceruloplasmin expression
GO:0022618: ribonucleoprotein complex assembly
GO:0002181: cytoplasmic translation
CORUM:3047: Parvulin-associated pre-rRNP complex
GO:0034101: erythrocyte homeostasis
GO:0009895: negative regulation of catabolic process
ko00190: Oxidative phosphorylation
R-MMU-2173795: Downregulation of SMAD2/3:SMAD4 transcriptional activity
R-MMU-195721: Signaling by WNT
GO:0034248: regulation of cellular amide metabolic process
GO:0072075: metanephric mesenchyme development
GO:0051129: negative regulation of cellular component organization
GO:0010942: positive regulation of cell death
GO:0061458: reproductive system development
GO:0040008: regulation of growth
GO:1902532: negative regulation of intracellular signal transduction
ko04120: Ubiquitin mediated proteolysis
GO:0000302: response to reactive oxygen species
GO:0071333: cellular response to glucose stimulus
GO:0034063: stress granule assembly
-log10(P)
G
Bmpr1a
Smad3
Expression Level
PD0
PD0-DEHP
PD3
PD3-DEHP
H
PD3
PD3-DEHP
BMPR1A
SMAD3
GAPDH
60 kDa
58 kDa
37 kDa
BMPR1A
SMAD3
N=3
**
Relative level
PD3
PD3-DEHP

Figure S8

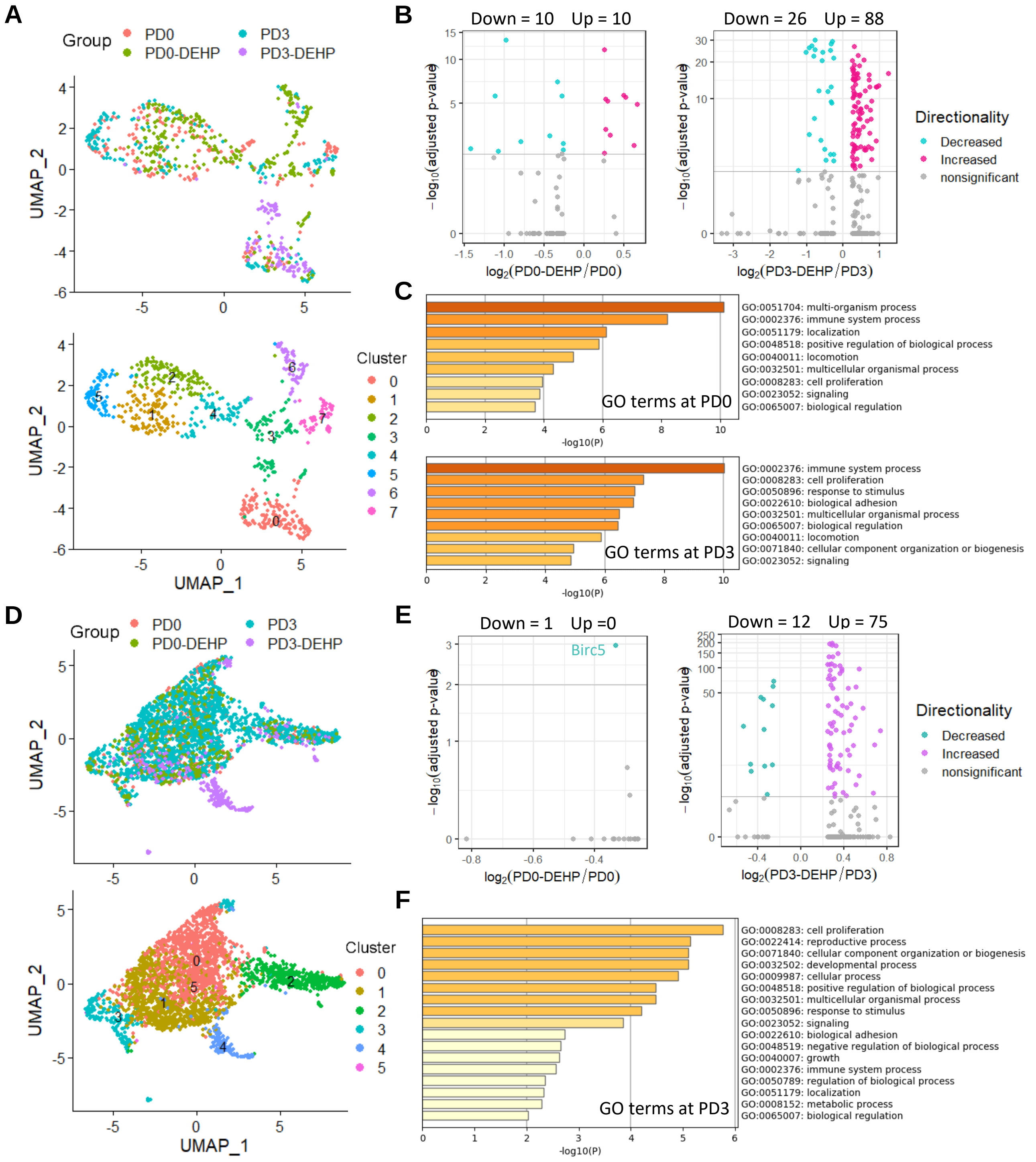
A
Group PD0 PD3 PD0-DEHP PD3-DEHP
UMAP_2
UMAP_1
Cluster 0 1 2 3 4 5 6 7
B
Down = 10 Up = 10
Down = 26 Up = 88
−log10(adjusted p-value)
log2(PD0-DEHP/PD0)
log2(PD3-DEHP/PD3)
Directionality
Decreased
Increased
nonsignificant
C
GO:0051704: multi-organism process
GO:0002376: immune system process
GO:0051179: localization
GO:0048518: positive regulation of biological process
GO:0040011: locomotion
GO:0032501: multicellular organismal process
GO:0008283: cell proliferation
GO:0023052: signaling
GO:0065007: biological regulation
GO terms at PD0
-log10(P)
GO:0002376: immune system process
GO:0008283: cell proliferation
GO:0050896: response to stimulus
GO:0022610: biological adhesion
GO:0032501: multicellular organismal process
GO:0065007: biological regulation
GO:0040011: locomotion
GO:0071840: cellular component organization or biogenesis
GO:0023052: signaling
GO terms at PD3
-log10(P)
D
Group PD0 PD3 PD0-DEHP PD3-DEHP
UMAP_2
UMAP_1
Cluster 0 1 2 3 4 5
E
Down = 1 Up =0
Birc5
Down = 12 Up = 75
−log10(adjusted p-value)
log2(PD0-DEHP/PD0)
log2(PD3-DEHP/PD3)
Directionality
Decreased
Increased
nonsignificant
F
GO:0008283: cell proliferation
GO:0022414: reproductive process
GO:0071840: cellular component organization or biogenesis
GO:0032502: developmental process
GO:0009987: cellular process
GO:0048518: positive regulation of biological process
GO:0032501: multicellular organismal process
GO:0050896: response to stimulus
GO:0023052: signaling
GO:0022610: biological adhesion
GO:0048519: negative regulation of biological process
GO:0040007: growth
GO:0002376: immune system process
GO:0050789: regulation of biological process
GO:0051179: localization
GO:0008152: metabolic process
GO:0065007: biological regulation
GO terms at PD3
-log10(P)
